# Clinical Characteristics of Patients with Myocarditis following COVID-19 mRNA Vaccination: A Systematic Review and Meta-Analysis

**DOI:** 10.3390/jcm11154521

**Published:** 2022-08-03

**Authors:** Reem H. Matar, Rania Mansour, Hayato Nakanishi, Karen Smayra, Joe El Haddad, Dilip K. Vankayalapati, Rohan Suresh Daniel, Danijel Tosovic, Christian A. Than, Mohamad H. Yamani

**Affiliations:** 1 Faculty of Medicine, St George’s University of London, London SW17 0RE, UK; mansour.r@live.sgul.ac.cy (R.M.); nakanishi.h@live.sgul.ac.cy (H.N.); smayra.k@live.sgul.ac.cy (K.S.); elhaddad.j@live.sgul.ac.cy (J.E.H.); vankayalapati.d@live.sgul.ac.cy (D.K.V.); sureshdaniel.r@live.sgul.ac.cy (R.S.D.); c.than@uq.edu.au (C.A.T.); 2 Faculty of Medicine, University of Nicosia Medical School, University of Nicosia, Nicosia 2417, Cyprus; 3 Division of Gastroenterology and Hepatology, Mayo Clinic, Rochester, MN 55905, USA; 4 School of Biomedical Sciences, The University of Queensland, St. Lucia, Brisbane 4072, Australia; d.tosovic@uq.edu.au; 5 Department of Cardiovascular Medicine, Mayo Clinic, Jacksonville, FL 32224, USA; yamani.mohamad@mayo.edu

**Keywords:** vaccine, vaccination, mRNA, myocarditis, COVID-19

## Abstract

COVID-19 mRNA vaccinations have recently been implicated in causing myocarditis. Therefore, the primary aim of this systematic review and meta-analysis was to investigate the clinical characteristics of patients with myocarditis following mRNA vaccination. The secondary aims were to report common imaging and laboratory findings, as well as treatment regimes, in these patients. A literature search was performed from December 2019 to June 2022. Eligible studies reported patients older than 18 years vaccinated with mRNA, a diagnosis of myocarditis, and subsequent outcomes. Pooled mean or proportion were analyzed using a random-effects model. Seventy-five unique studies (patient *n* = 188, 89.4% male, mean age 18–67 years) were included. Eighty-six patients had Moderna vaccines while one hundred and two patients had Pfizer-BioNTech vaccines. The most common presenting symptoms were chest pain (34.5%), fever (17.1%), myalgia (12.4%), and chills (12.1%). The most common radiologic findings were ST-related changes on an electrocardiogram (58.7%) and hypokinesia on cardiac magnetic resonance imaging or echocardiography (50.7%). Laboratory findings included elevated Troponin I levels (81.7%) and elevated C-reactive protein (71.5%). Seven patients were admitted to the intensive care unit. The most common treatment modality was non-steroid anti-inflammatory drugs (36.6%) followed by colchicine (28.5%). This meta-analysis presents novel evidence to suggest possible myocarditis post mRNA vaccination in certain individuals, especially young male patients. Clinical practice must therefore take appropriate pre-cautionary measures when administrating COVID-19 mRNA vaccinations.

## 1. Introduction

In early December 2019, the first case of coronavirus was identified in Wuhan, China [1,2]. On 11 March 2020, COVID-19 was officially declared a global pandemic by the World Health Organization (WHO) [3]. Since then, COVID-19 has affected 542,188,789 individuals globally and taken more than 6,329,275 lives [4]. Moreover, the disease has mutated exponentially, causing multiple variants and exceptional damage [5]. As of now, COVID-19 continues to be rampant, burdening healthcare systems globally whilst consistently instilling public fear through sporadic surges in cases with the looming threat of additional waves forthcoming [6]. Therefore, substantial efforts have been directed to combat and manage this disease.

Currently, three companies (Pfizer-BioNTech, Moderna, and Johnson and Johnson) have manufactured vaccines that have been approved for emergency use by the U.S. Food and Drug Administration (FDA) based on double-blinded, randomized, controlled clinical trials [7]. Two of these vaccines are messenger RNA-based (mRNA) vaccines—BNT162b2 (Pfizer-BioNTech) and mRNA-1273 (Moderna)—that encode the spike protein antigen of SARS-CoV-2, encapsulated in lipid nanoparticles [8]. Both mRNA-based vaccines are considered safe for public implementation and offer up to 94% protection from COVID-19 infection [9]. However, considering the rapid response to vaccine development and clinical administration, there have been ongoing reports of vaccine-induced adverse events in relation to mRNA vaccines. To date, more than 500,000 adverse events have been reported to the Vaccine Adverse Events Reporting System (VAERS) [10]. 

Approximately 11,833,638,209 vaccines have been administered around the world, roughly half being mRNA associated vaccines. Shortly after receiving vaccine doses, patients began reporting various vaccine-related side effects. For instance, a study by Teo et al. looked at the safety and efficacy of the Pfizer BioNTech and Moderna vaccination in young adolescents aged 18 years and above, demonstrating vaccine-related side effects such as fatigue, headache, pain, and chills [11]. Recently, myocarditis has come to light as a possible side effect. The first few cases of myocarditis following mRNA vaccination were reported at the end of February in Israel [12]. Since then, more cases of myocarditis have surfaced in the literature. In response, the Centers for Disease Control and Prevention (CDC) set up an emergency meeting with its advisory committee in June 2021 to discuss the rise in the rate of confirmed cases of myocarditis following mRNA COVID-19 vaccination in people under 30 years of age [13]. In addition, the VAERS has received more than 1000 adverse events reports of myocarditis following COVID-19 mRNA vaccination in adults aged 18 and over [10]. It should be noted however that post-vaccination symptomology cannot currently be differentiated from an active COVID-19 infection. As such, routine COVID-19 testing should continue in those with post-vaccination symptoms to prevent community spread [14].

On 10 May 2021, the FDA approved the emergency use authorization of the Pfizer-BioNTech COVID-19 Vaccine in children aged 12–15 years [15]. Unfortunately, cases of myocarditis have also been reported in the pediatric population [16]. As more individuals continue to get vaccinated, specifically children, there is an urgent need to answer questions regarding the safety of the mRNA vaccinations (Pfizer BioNTech and Moderna), specifically pertaining to its association with increased risk of myocarditis. Although the results available in the literature may be sparse, it is important to recognize the urgency and time-sensitivity of this issue. Therefore, this systematic review and meta-analysis aims to provide insight into the clinical characteristics of patients diagnosed with myocarditis following COVID-19 mRNA vaccination. Further insight into the common imaging and laboratory findings, as well as treatment modalities, were a secondary aim of this meta-analysis. 

## 2. Methods

### 2.1. Search Strategy and Data Sources

A comprehensive search of several databases from 1 December 2019, as the first case of COVID-19 was identified at this time, to 20 June 2022 was conducted and limited to English language only. The databases included Ovid MEDLINE(R) and Epub Ahead of Print, In-Process & Other Non-Indexed Citations, Daily, Ovid EMBASE, Ovid Cochrane Central Register of Controlled Trials, Ovid Cochrane Database of Systematic Reviews, and Scopus. The search strategy was designed and conducted by a medical reference librarian. Controlled vocabulary supplemented with keywords was used to search for studies describing myocarditis following COVID-19 vaccination. The actual strategy listing all search terms used and how they are combined is available in Supplementary Item S1.

### 2.2. Eligibility Criteria and Quality Assessment

Eligible studies must have met all the following inclusion criteria: (1) Participants must be older than 18 years, vaccinated with one of the approved vaccinations; (2) Diagnosed with myocarditis; and (3) Reported clinical characteristics of patients. The methodological quality of each study was independently evaluated by two authors (RHM and CAT) using the methodological quality and synthesis of case series and case reports as has been previously described within literature [17].

## 3. Statistical Analysis

Means of continuous variables and rates of binary variables were pooled using the random-effects model, and the generic inverse variance method of DerSimonian, Laird [18]. Proportions underwent logit transformation prior to meta-analysis. The heterogeneity of effect size estimates across the studies was quantified using the Q statistic and the I^2^ index (*p* < 0.10 was considered significant). A value of I^2^ of 0–25% indicates minimal heterogeneity, 26–50% moderate heterogeneity, and 51–100% substantial heterogeneity. Data analysis was performed using Open Meta analyst software (CEBM, Brown University, Providence, RI, USA). 

## 4. Results

### 4.1. Study Selection and Characteristics

The initial search yielded 268 potentially relevant articles from which 75 unique studies involving 188 patients met eligibility criteria [16,19,20,21,22,23,24,25,26,27,28,29,30,31,32,33,34,35,36,37,38,39,40,41,42,43,44,45,46,47,48,49,50,51,52,53,54,55,56,57,58,59,60,61,62,63,64,65,66,67,68,69,70,71,72,73,74,75,76,77,78,79,80,81,82,83,84,85,86,87,88,89,90,91,92]. The details of the study selection process are depicted in Supplementary Item S2. The baseline characteristics of the included studies are comprehensively described in Table 1. The age ranged from 18–67 years, of which 168 patients were males.

### 4.2. Risk of Bias

Results of the quality assessment of all included studies are shown in Supplementary Table S1. All the case series were judged to be of adequate quality. The patients appeared to represent the whole experience of the investigator and the exposure and outcome were adequately ascertained, and the length of follow-up was adequate. 

### 4.3. Clinical Characteristics

The clinical characteristics of the patients are shown in Table 2. Of the 188 patients, 102 patients received the Pfizer BioNTech vaccine while 86 patients received the Moderna vaccine. Among the overall population, there were 95 reports of comorbidities; frequently reported illnesses included hypertension, hyperlipidaemia, and hypothyroidism. Only three patients had history of relevant cardiac problems which included right bundle branch block, left ventricular cardiomyopathy, and congestive heart failure. Out of the 188 patients, 11 patients had a previous history of COVID-19 infection. Additionally, 25 patients had symptoms of myocarditis following the first dose of the mRNA vaccine, 154 patients had symptoms of myocarditis following the second dose of the mRNA vaccine, and 9 patients had symptoms of myocarditis following the third dose of the mRNA vaccine. The pooled mean time from symptom onset following vaccination to admission was 2.488 days (95% CI: 1.852; 3.125, I^2^ = 73.64%).

**Figure 1:**
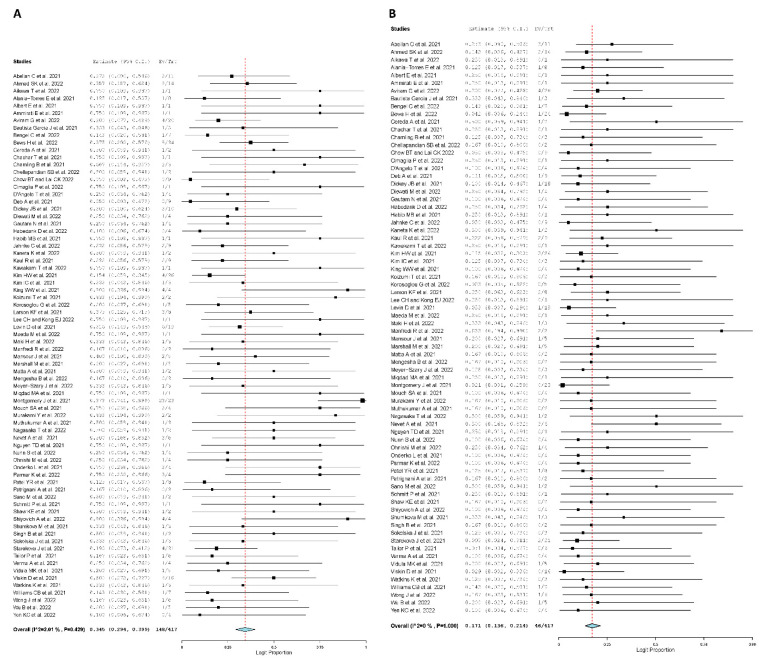

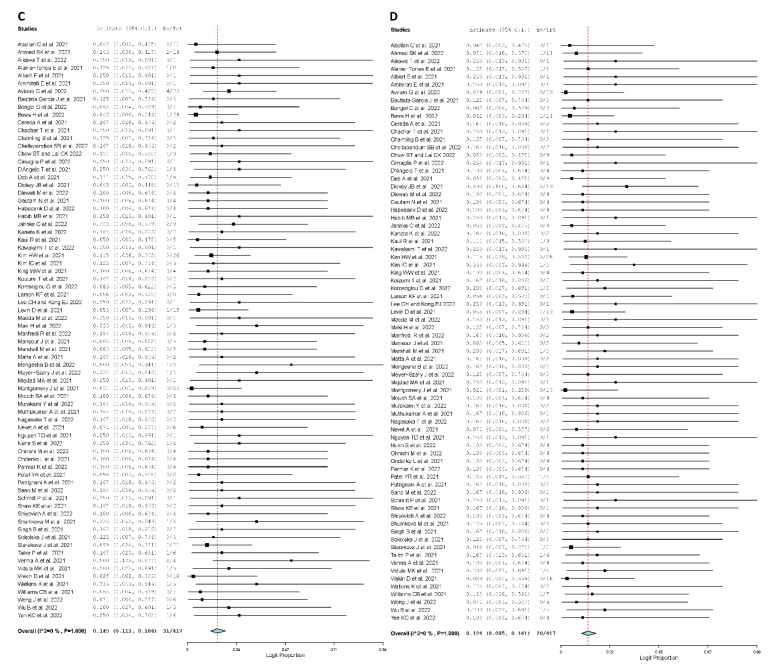
Forest plot of presenting symptoms. (**A**), Chest pain. (**B**), Fever. (**C**), Dyspnea. (**D**) Myalgia [16,19,20,21,22,23,24,25,26,27,28,29,30,31,32,33,34,35,36,37,38,39,40,41,42,43,44,45,46,47,48,49,50,51,52,53,54,55,56,57,58,59,60,61,62,63,64,65,66,67,68,69,70,71,72,73,74,75,76,77,78,79,80,81,82,83,84,85,86,87,88,89,90,91,92].

At time of admission, a total of 417 symptoms were reported. Among those symptoms, the most frequent symptoms were chest pain with a pooled rate of 34.5% (95% CI: 0.294, 0.399; I^2^ = 2.01%), fever with a pooled rate of 17.1% (95% CI: 0.136, 0.214; I^2^ = 0%), dyspnea with a pooled rate of 14.5% (95% CI:0.113, 0.184; I^2^ = 0%), myalgia with a pooled rate of 12.4% (95% CI: 0.095, 0.161; I^2^ = 0%), and chills with a pooled rate of 12.1% (95% CI: 0.092, 0.157; I^2^ = 0%) (Figure 1).

### 4.4. Radiological and Laboratory Findings

Table 3 and Table 4, respectively, demonstrate the pertinent radiologic and laboratory findings on admission. It should be noted that all findings were based on imaging and laboratory reporting numbers only, and not number of patients. A total of six-hundred-twenty-one imaging scans were performed at the time of admission, of which 185 were electrocardiograms (ECG), 133 were Cardiac MRIs (cMRI), 144 were Echocardiograms (ECHO), 33 were Cardiac CTs, 58 were Coronary Angiograms, 46 were Chest-X rays (CXR), 9 Chest CTs, and 13 were a cardiac biopsy.

**Figure 2:**
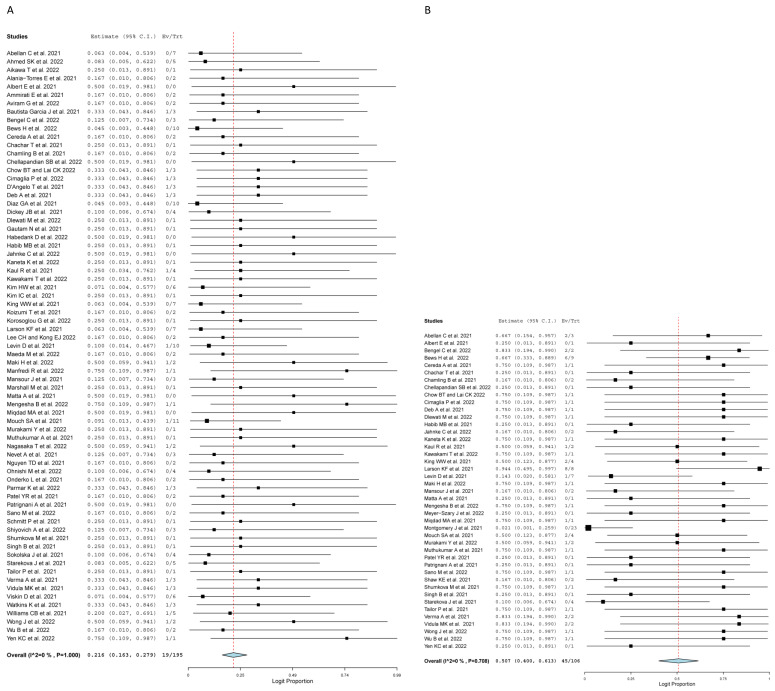

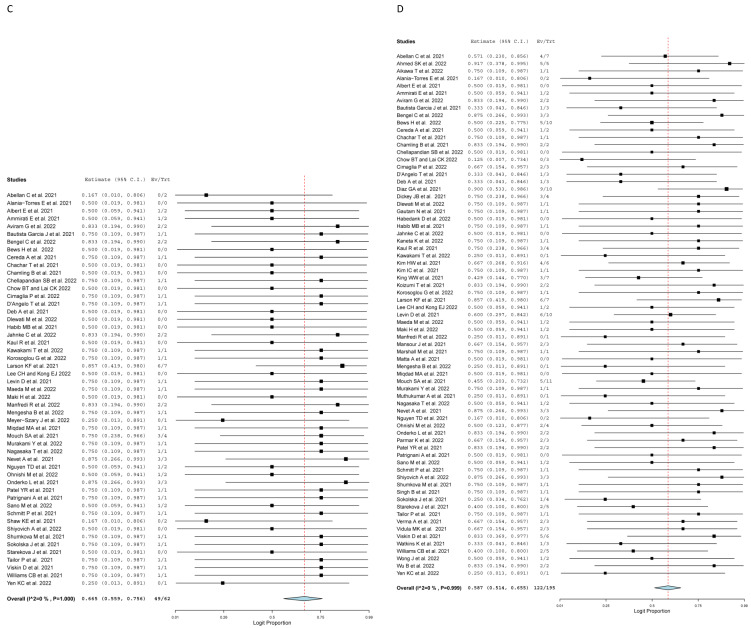
Forest plot of radiological findings at time of admission. (**A**) Arrhythmia. (**B**) Hypokinesis. (**C**) Myocardial Edema. (**D**) ST Changes [16,19,20,21,22,23,24,25,26,27,28,29,30,31,32,33,34,35,36,37,38,39,40,41,42,43,44,45,46,47,48,49,50,51,52,53,54,55,56,57,58,59,60,61,62,63,64,65,66,67,68,69,70,71,72,73,74,75,76,77,78,79,80,81,82,83,84,85,86,87,88,89,90,91,92].

One hundred eighty-five ECG records were obtained at the time of admission, and a total of 188 abnormalities were reported, of which 122 were ST-related changes (58.7%; 95% CI: 0.514, 0.655; I^2^ = 0%). Of the 122 ST-related changes, 96 were ST-elevations (68.8%; 95%CI: 0.605, 0.761; I^2^ = 0%) (Figure 2). Moreover, twenty-two T wave changes were identified (22.5%; 95% CI: 0.172, 0.288; I^2^ = 0%); likewise, twenty-three PR changes were identified (23.1%; 95% CI: 0.179, 0.292; I^2^ = 0%), of which twenty PR-segment depression were identified (56.9%; 95% CI: 0.460, 0.672; I^2^ = 0%) and six QRS complex changes were identified (17.3%; 95% CI: 0.127, 0.230; I^2^ = 0%). Arrhythmias were identified in nineteen ECGs (21.6%; 95% CI: 0.163, 0.279; I^2^ = 0%) (Figure 2). Additionally, Montgomery et al. reported 19 cases of abnormality including ST elevations, T-wave inversion, and non-specific ST changes. 

**Figure 3:**
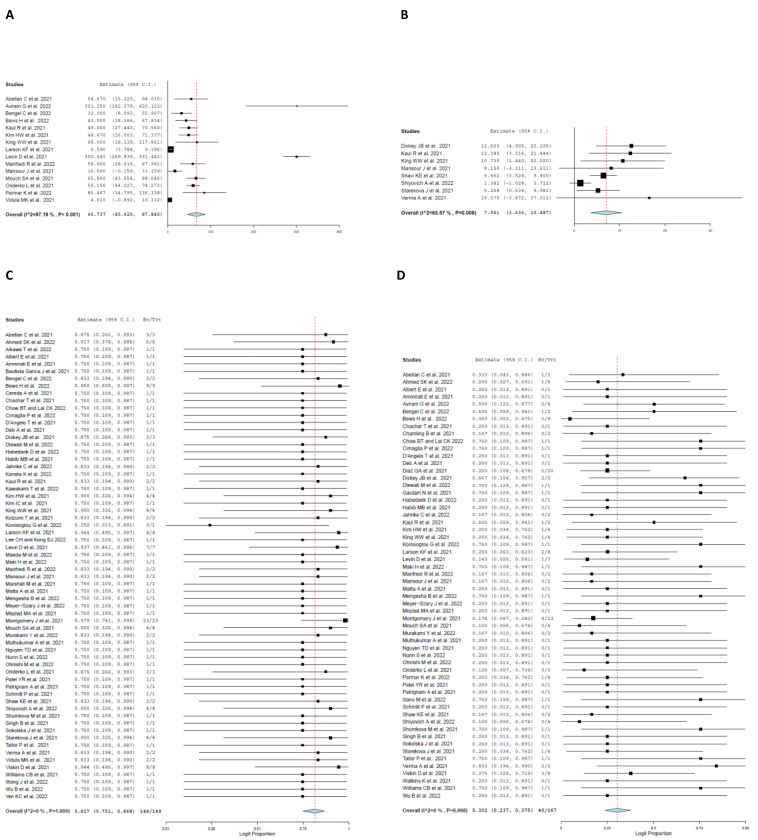
Forest plot of laboratory findings at time of admission. (**A**) C Reactive Protein. (**B**) Cardiac Troponin-I. (**C**) Elevated Troponin. (**D**) Left Ventricular Ejection Fraction Less Than 50% [16,19,20,21,22,23,24,25,26,27,28,29,30,31,32,33,34,35,36,37,38,39,40,41,42,43,44,45,46,47,48,49,50,51,52,53,54,55,56,57,58,59,60,61,62,63,64,65,66,67,68,69,70,71,72,73,74,75,76,77,78,79,80,81,82,83,84,85,86,87,88,89,90,91,92].

Among 133 cMRI scans, 62 cases of cardiac edema were reported, of which 49 were myocardial edema, eight were subepicardial edema, and five were pericardial/epicardial edema (Figure 3). Furthermore, there were 53 reported cases of regional wall motion abnormality, of which 45 cases were of hypokinesia (50.7%; 95% CI: 0.400, 0.613; I^2^ = 0%), two cases of cardiomegaly, and twenty-five cases of pericardial effusion. Left Ventricular Ejection Fraction (LVEF) was measured in 167 patients at the time of admission with either ECHO or cMRI, of which 40 patients had LVEF lower than fifty percent (23.7%; 95% CI: 0.237, 0.375; I^2^ = 0%) (Figure 3).

One hundred forty-four ECHO imaging were reported at the time of admission and 62 abnormalities were observed besides reduced LVEF, including 31 cases of hypokinesis, seven cases of pericardial effusion, one case of pericardium hyperechogenicity, one case of a mildly dilated left atrium, six cases of impaired speckled strain, one case of global longitudinal deformation, three enlarged LVs, two enlarged RVs, one case of LV hypertrophy, one case of dilated inferior vena cava (IVC), two cases of grade 1 diastolic dysfunction, one case of RV systolic dysfunction, four cases of LV systolic dysfunction, and one case of grade 2 diastolic dysfunction.

Thirty-three Cardiac CT scans were reported, and the result showed no marks of acute aortic syndrome or coronary artery disease. Two Cardiac CT scans reported a coronary artery calcium score of zero. Six Cardiac CT scans reported delayed iodine enhancement at the 7 min scan, with typical distribution of myocarditis. Additionally, fifty-eight coronary angiogram scans were performed at the time of admission, and no significant primary vessel stenosis, flow abnormalities, visible thrombus, or acute coronary syndrome were observed. Three scans had very mild non-obstructive coronary artery disease. One scan had an incidental coronary artery fistula finding. In addition, one scan had a 50% occlusion of the posterior descending artery and 90% stenosis of one of the two right posterolateral artery branches. 

A total of 46 CXRs were performed at the time of admission. Five scans demonstrated evidence of pulmonary edema. The remainder of the CXRs were reported as negative or insignificant. Finally, 13 patients underwent a cardiac biopsy, three of which demonstrated no myocardial infiltrate, two biopsies demonstrated myocardial edema, one biopsy demonstrated myocyte hypertrophy, one biopsy demonstrated myocyte vacuolization, one biopsy demonstrated interstitial fibrosis, and seven biopsies demonstrated an inflammatory infiltrate predominantly composed of T-cells and macrophages, mixed with eosinophils, B cells, and plasma cells.

On admission, the pooled mean cTroponin I was 7.061 ng/mL (95% CI: 3.636, 10.487; I^2^ = 63.57%). Elevated troponin levels were present in 148 patients with a pooled proportion of 81.7% (95% CI: 0.751, 0.868; I^2^ = 0%). Additionally, the pooled mean C-reactive protein (CRP) was 66.737 mg/l (95% CI: 45.629, 87.845; I^2^ = 97.19%). Elevated CRP levels were present in patients with a pooled proportion of 71.5% (95% CI: 0.628, 0.788; I^2^ = 0%). The pooled mean of LVEF was 55.587% (95% CI: 54.151, 57.023, I^2^ = 45.25%). 

### 4.5. Treatment and Clinical Course

**Figure 4:**
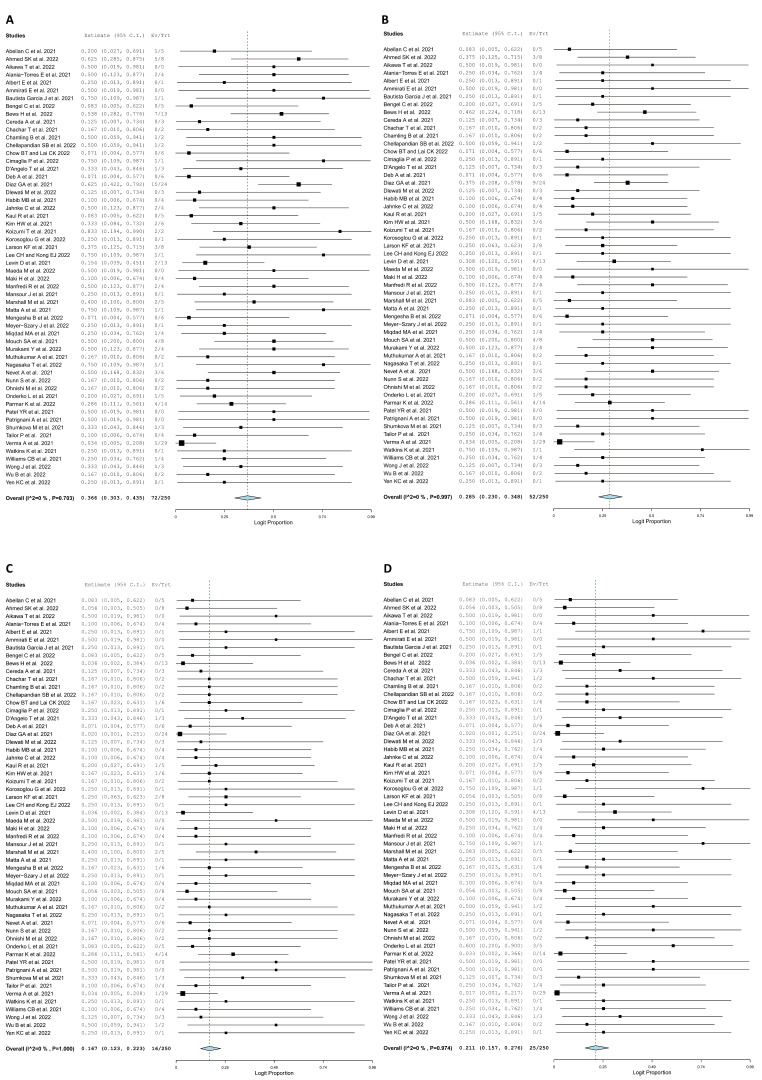
Forest plot of treatment modality. (**A**) NSAID. (**B**) Colchicine. (**C**) Steroids. (**D**) Beta-blocker [16,19,20,21,22,23,24,25,26,27,28,29,30,31,32,33,34,35,36,37,38,39,40,41,42,43,44,45,46,47,48,49,50,51,52,53,54,55,56,57,58,59,60,61,62,63,64,65,66,67,68,69,70,71,72,73,74,75,76,77,78,79,80,81,82,83,84,85,86,87,88,89,90,91,92].

**Figure 5:**
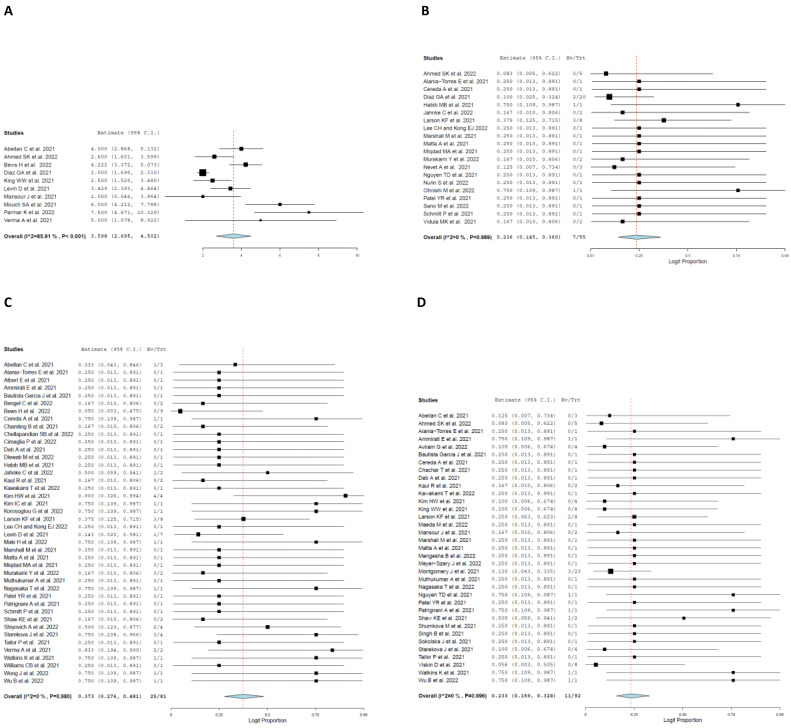
Forest plot of clinical course and other pertinent clinical findings. (**A**) Length of hospital stay. (**B**) Intensive care unit admission. (**C**) Pericardial effusion. (**D**) Prior COVID infection [16,19,20,21,22,23,24,25,26,27,28,29,30,31,32,33,34,35,36,37,38,39,40,41,42,43,44,45,46,47,48,49,50,51,52,53,54,55,56,57,58,59,60,61,62,63,64,65,66,67,68,69,70,71,72,73,74,75,76,77,78,79,80,81,82,83,84,85,86,87,88,89,90,91,92].

Table 5 shows information about clinical course and treatment details of patients. Fifty-five patients presented to the emergency room (73.2%; 95% CI: 0.627, 0.817, I^2^ = 0%), while seven patients were admitted to the intensive care unit (ICU) (23.6%; 95% CI: 0.145, 0.360, I^2^ = 0%). Patients received various treatment modalities over the course of their stay (Figure 4). Seventy-two non-steroidal anti-inflammatory (NSAIDs) were administered to patients with a pooled rate of 36.6% (95% CI: 0.303, 0.435, I^2^ = 0) and fifty-two colchicine regimes were administered to patients with a pooled rate of 28.5% (95% CI: 0.230, 0.348, I^2^ = 0%). Sixteen steroids were administered to patients with a pooled rate of 16.7% (95% CI: 0.123, 0.223, I^2^ = 0%). Lastly, twenty-five beta-blocker medications were administered with a pooled rate of 21.1% (95% CI: 0.157, 0.276, I^2^ = 0%). Only one patient required supplemental oxygen therapy. Additionally, none of the patients required mechanical circulatory support. One patient died due to cardiogenic shock. Lastly, sixty-two patients were followed-up in clinic after diagnosis of myocarditis. The length of hospital stay was an average of 3.598 days (95% CI: 2.695, 4.502, I^2^ = 85.91%) (Figure 5). 

## 5. Discussion

The primary aim of this systematic review and meta-analysis was to investigate clinical characteristics of myocarditis among patients following mRNA COVID-19 vaccination. A total of 75 studies reporting 188 patients with myocarditis post-COVID-19 vaccination were found. Within this meta-analysis, the following has been supported regarding characteristics of patients diagnosed with myocarditis post-vaccination: (1) chest pain is the most common symptomatic finding among diagnosed patients; (2) myocarditis is more prevalent among males compared to females post-vaccination; (3) myocarditis appears to be more likely to occur following the second dose of mRNA COVID-19 vaccination, yet can still occur following the first or third dose; (4) radiological findings of myocarditis appear to most commonly involve ST changes on electrocardiography and normal left ventricular ejection fractions (LVEF) on echocardiography or cardiac magnetic resonance imaging; (5) elevated troponin levels within patient serum appears to be a consistent finding among studies that report laboratory results. As such, the results of this study may not only provide insight into the clinical investigation and management of myocarditis but provides further evidence to warrant future investigations into individual eligibility for COVID-19 mRNA vaccines.

This meta-analysis identified the occurrence of 417 symptoms at admission among a total of 188 patients whose age ranged from 18 to 67 years. Of all patients, 102 had received the Pfizer-BioNTech vaccine, and 86 received the Moderna vaccine. Major symptoms reported in this meta-analysis, associated with diagnosis of myocarditis, were chest pain, fever, dyspnea, myalgia, and chills. This is consistent with literature on the common clinical features of myocarditis, in which an early systematic review identifying patients with COVID-19-associated myocarditis indicated similar symptom presentation [93]. 

In addition to the broad range in symptomology, it has been documented that the risk of developing myocarditis is greater for the male sex compared to the female sex [94,95]. Available research suggests that the elevated testosterone inherent to the male sex may enhance inflammation, subsequently aggravating the fibrosis associated with myocarditis and increasing the probability of developing chronic cardiovascular conditions such as cardiomyopathy, as is often reported following myocarditis [96]. This could explain the similar trends seen following mRNA COVID-19 vaccination, as 89.4% of the current study’s sample size were male (Table 1). It is thus imperative to investigate this association further to ensure the development of evidence-based clinical guidelines regarding eligibility for mRNA COVID-19 vaccines, particularly among males.

The pooled onset of myocarditis symptoms appearing more predominantly after the second dose of mRNA COVID-19 vaccination is in line with nation-wide electronic medical record assessments made by the FDA and the CDC [97]. Development of myocarditis following vaccination is not uncommon, as prior extensive reporting following vaccination for smallpox, anthrax, trivalent influenza and influenza type B, hepatitis B, and others has been documented [98,99,100,101]. The pathophysiological mechanism causing post-vaccine myocarditis is unclear. At present, it is hypothesized that those with genetic predispositions for immune hyperresponsiveness may have activation of an aberrant innate and acquired immune response to the mRNA vaccines. This in turn may cause activation of proinflammatory cascades and immunologic pathways that could contribute to myocarditis as part of a systemic reaction in certain individuals [102].

Two autopsy case reports of endomyocardial biopsies taken from patients who had received the tetanus and smallpox vaccines have documented the presence of lymphocytic and eosinophilic infiltration surrounding the necrotic myocardium [103,104]. Collectively, such data suggest a maladaptive immune-mediated injury or hypersensitivity reaction [55]. Given that two doses of the mRNA vaccines are required to elicit adequate neutralizing antibody titers and antigen specific responses [105,106], the suggested pathophysiological mechanism may explain the increased frequency of myocarditis following vaccination with the second dose, as seen in 155 of 188 patients in this meta-analysis. The gold standard to ascertain the etiology of post-vaccination myocarditis is an endomyocardial biopsy [107,108]. However, an endomyocardial biopsy may not be indicated in hemodynamically stable myocarditis with preserved systolic function [109]. Within this meta-analysis, only 11 studies reported evaluation of a cardiac biopsy, two of which did not demonstrate myocardial infiltrate [59,60]. Moreover, as patients often improve rapidly with treatment, conducting a cardiac biopsy may not be relevant [110]. This presents an opportunity to explore alternative methods to determine the cause of post-vaccination myocarditis, which would be a crucial first step in the appropriate management of hospitalized patients.

The limitation of endomyocardial biopsies has enabled a more significant role for laboratory studies and radiological investigations in the diagnosis of myocarditis. Elevated cardiac enzymes, and in particular troponin levels, are a strong indicator for cardiac myonecrosis and have been the hallmark among patients with COVID-19-related myocarditis [111]. In a cohort of 386 patients with myocarditis, 100% of patients were found with elevated Troponin T levels and 99% of patients were found with abnormal values of acute phase reactants, namely erythrocyte sedimentation rate (ESR) or C-reactive protein (CRP) [112]. This meta-analysis presents similar findings. Elevated troponin levels were found in 81.7% of patients who had been tested, whereas abnormal CRP was prevalent in 71.5% of patients who had been tested. It must be noted that the absence of elevated levels of troponin or acute phase reactants, however, does not rule out myocarditis [113]. As such, imaging techniques have been applied in the diagnosis of myocarditis to rapidly exclude coronary ischemia and other causes of heart failure. The predominant echocardiographic findings of normal LVEF (67.5% patients) demonstrated throughout this analysis are supported by studies emphasizing the essential role of early echocardiography in establishing the diagnosis and severity of cardiac compromise in myocarditis [114,115]. The American Heart Association has also specified ECG outcomes of myocarditis to include ST elevations, low QRS voltage, and PR depression [113]. In line with this data, the most predominant ECG finding from patients in this meta-analysis were ST changes, found in 58.7% of patients. 

Tissue-level pathologies consistent with myocarditis, such as acute necrosis, chronic fibrosis, and myocardial edema, can be uniquely visualized via gadolinium contrast-enhanced cardiac magnetic resonance (CMR) [116,117]. Traditional guidelines for myocarditis, commonly known as the Lake Louise Criteria, recommend considering two of three CMR tissue characterization criteria when diagnosing myocarditis [113]; this has demonstrated a diagnostic accuracy of 79% [118]. In this meta-analysis, multiple studies relied on Cardiac MRI alone for diagnosis of post-vaccine myocarditis, moreover, other studies relied on Lake Louise Criteria, and a small number of studies utilized a combination of laboratory and non-invasive data. However, a cohort study aimed at validating the 2018 Lake Louise Criteria recommend multiparametric CMR for the diagnosis of myocarditis [119]. Multiple studies support the diagnostic value of myocardial T1 mapping in detecting myocarditis based on results yielding sensitivity in the range of 78–89% and specificity in the range of 86–96% [119,120,121]. The mechanisms resulting in an increase of myocardial T1 in acute myocarditis include intracellular and extracellular edema, vasodilation, acute necrosis, and hyperemia [117,122,123]. However, chronic myocarditis and diseases accompanied by myocardial fibrosis also present with increased myocardial T1 relaxation times [124,125,126]. Hence, myocardial T1 times are not specific to acute myocarditis, as is the case of post-vaccination myocarditis. Although, myocardial T2 mapping is also a sensitive parameter for myocardial edema as it can uniquely differentiate between acute and healing stages of myocarditis [127]. Thus, T2 mapping more accurately detects inflammatory changes compared to T1 mapping in cardiac investigations [128]. However, both T1 and T2 mapping techniques lack standardized or consistent cut off values for the direct diagnosis of acute myocarditis alone [119]. As such, results must be interpreted based on the presence of additional clinical features and not of cardiac MRI alone. Despite concerns regarding standardized values for myocardial T1 and T2 mapping in acute myocarditis, studies have shown that the implementation of quantitative CMR parameters, namely T1 and T2 mapping with LGE imaging, drastically improves the diagnostic performance and accuracy of cardiac MRI [125,126,129]. However, in this meta-analysis, T1 and T2 mapping times were generally not reported and therefore the data could not be pooled due to lack of information from studies. The reason for conducting T1 vs. T2 mapping is uncertain, yet likely to be based on institution-specific guidelines. Therefore, this meta-analysis cannot conclude on comparisons of these mapping modalities. As such, these results warrant a systematic review on the role of multiparametric CMR and the role of clinical features in the diagnosis of myocarditis; this could largely aid in the development of best-practice guidelines for the investigation and diagnosis of myocarditis.

Considering the rapid progression of the COVID-19 virus, the development of variants, and emergency authorization of the Pfizer-BioNTech and Moderna vaccines, the limitations of this current systematic review and meta-analysis must be addressed. Most significantly is the lack of high-quality data in the included studies. Due to the reliance on subjective measures such as comorbidities, medical histories, and family history, as well as the urgent timeline for data reporting, several studies presented with incomplete documentation of quantitative outcomes such as laboratory values and details from radiographic investigations. Additional parameters not fully documented included epidemiological history, clinical outcomes upon reported follow-up, and in only a few instances, the specific cardiac enzymes evaluated. Follow-up was reported in 62 patients, however, due discrepancies in follow-up methods, a detailed discussion on outcomes following myocarditis development was precluded. Furthermore, this analysis included a limited number of patients derived from a relatively limited number of case reports and case series, most of which were only issued a few weeks prior to conducting this meta-analysis. As such, it is difficult to correlate certain imaging abnormalities, such as a dilated left atrium, with myocarditis. Nevertheless, these findings were still reported due to their nature as imaging findings. Additionally, a causal link between the mRNA COVID-19 vaccines and the development of myocarditis in certain individuals can only be suggested and not concluded. For similar reasons, conclusions regarding the eligibility criteria of mRNA COVID-19 vaccinations should be avoided. Lastly, due to the global nature of this virus, the expedited administration of vaccines, this meta-analysis may have overlooked recently published studies, especially in languages other than English. 

## 6. Conclusions

This meta-analysis presents evidence suggesting the development of myocarditis following mRNA-COVID-19 vaccination in certain individuals. Based on the data of this meta-analysis, males appear to be the predominant group affected by post-vaccination myocarditis, yet it has also been shown that females are as susceptible. In addition, the frequency and severity of symptoms, as well as the prevalence of myocarditis, appear to be greatest following the second dose of the vaccine compared to the first dose. Accurate identification of acute myocarditis is shown to be difficult given the non-specific and broad symptomology, the time-sensitive nature of conducting gold-standard diagnostic techniques such as endomyocardial biopsies, and the uncertain pathogenesis of disease. As such, further investigations are needed to understand the pathophysiological mechanisms underlying myocarditis following mRNA COVID-19 vaccination. Such clarifications may identify whether modifications to the eligibility criteria for the mRNA COVID-19 vaccinations are required. As of now, clinical practice must take appropriate pre-cautionary measures when administrating mRNA COVID-19 vaccinations. This could involve screening for pre-existing hypersensitive reactions and pre-emptive preparation of myocarditis treatment modalities upon vaccination.

## Figures and Tables

**Table 1:** Baseline Characteristics of Included Studies [16,19,20,21,22,23,24,25,26,27,28,29,30,31,32,33,34,35,36,37,38,39,40,41,42,43,44,45,46,47,48,49,50,51,52,53,54,55,56,57,58,59,60,61,62,63,64,65,66,67,68,69,70,71,72,73,74,75,76,77,78,79,80,81,82,83,84,85,86,87,88,89,90,91,92].

Author, Year	Country	Study Design	No. of Subjects (n)	Gender		Mean Age (year)		Vaccine Received (n)	
				Male	Female	Mean	SD	Pfizer BioNTech	Moderna
Abellan C et al., 2021 [92]	Switzerland	Case Series	3	3	0	28.67	14.15	0	3
Ahmed SK, 2022 [91]	Iraq	Case Series	5	5	0	27.80	7.36	3	2
Aikawa T et al., 2022 [89]	Japan	Case Report	1	1	0	20.00	NA	0	1
Alania-Torres E et al., 2021 [90]	Spain	Case Report	1	1	0	28.00	NA	1	0
Albert E et al., 2021 [88]	USA	Case Report	1	1	0	24.00	NA	0	1
Ammirati E et al., 2021 [87]	Italy	Case Report	1	1	0	56.00	NA	1	0
Aviram G et al., 2022 [86]	Israel	Case Series	4	4	0	29.25	6.99	4	0
Bautista Garcia J et al., 2021 [85]	Spain	Case Report	1	1	0	39.00	NA	1	0
Bengel C et al., 2022 [84]	Germany	Case Series	2	2	0	21.50	2.12	0	2
Bews H et al., 2022 [83]	Canada	Case Series	9	8	1	23.89	5.35	2	7
Cereda A et al., 2021 [79]	Italy	Case Report	1	1	0	21.00	NA	1	0
Chachar T et al., 2021 [80]	Bahrain	Case Report	1	1	0	24.00	NA	1	0
Chamling B et al., 2021 [81]	Germany	Case Series	2	2	0	22.50	3.53	2	0
Chellapandian SB et al., 2022 [82]	Qatar	Case Report	1	1	0	22.00	NA	0	1
Chow BT and Lai CK, 2022 [77]	Canada	Case Report	1	0	1	45.00	NA	0	1
Cimaglia P et al., 2022 [78]	Italy	Case Report	1	1	0	24.00	NA	1	0
D’Angelo T et al., 2021 [76]	Italy	Case Report	1	1	0	30.00	NA	1	0
Deb A et al., 2021 [75]	USA	Case Report	1	1	0	67.00	NA	0	1
Diaz GA et al., 2021 [74]	USA	Retrospective Study	20	15	5	36.00	16.30	9	11
Dickey JB et al., 2021 [73]	US	Case Series	3	3	0	NR	NR	2	1
Dlewati M et al., 2022 [72]	USA	Case Report	1	1	0	48.00	NA	0	1
Gautam N et al., 2021 [71]	USA	Case Report	1	1	0	66.00	NA	1	0
Habedank D et al., 2022 [70]	Germany	Case Report	1	1	0	60.00	NA	0	1
Habib MB et al., 2021 [69]	Qatar	Case Report	1	1	0	37.00	NA	1	0
Jahnke C et al., 2022 [68]	Germany	Case Series	2	2	0	31.50	14.85	1	1
Kaneta K et al., 2022 [67]	Japan	Case Report	1	1	0	25.00	NA	0	1
Kaul R et al., 2021 [66]	USA	Case Series	2	2	0	24.50	4.95	1	1
Kawakami T et al., 2022 [65]	Japan	Case Report	1	0	1	45.00	NA	0	1
Kim HW et al., 2021 [64]	USA	Case Series	4	3	1	38.25	21.98	2	2
Kim IC et al., 2021 [61]	Korea	Case Study	1	1	0	24.00	NA	1	0
King WW et al., 2021 [63]	USA	Case Series	4	3	1	25.50	4.79	1	3
Koizumi T et al., 2021 [60]	Japan	Case Study	2	2	0	24.50	3.54	0	2
Korosoglou G et al., 2022 [62]	Germany	Case Report	1	1	0	21.00	NA	1	0
Larson KF et al., 2021 [59]	USA and Italy	Case Series	8	8	0	31.63	11.99	5	3
Lee CH and Kong EJ, 2022 [58]	South Korea	Case Report	1	1	0	22.00	NR	0	1
Levin D et al., 2021 [56]	Israel	Case Series	7	7	0	20.43	2.07	7	0
Maeda M et al., 2022 [19]	Japan	Case Report	1	1	0	29.00	NA	0	1
Maki H et al., 2022 [54]	Japan	Case Report	1	0	1	20.00	NA	0	1
Manfredi R et al., 2022 [57]	Italy	Case Series	2	2	0	21.50	4.95	1	1
Mansour J et al., 2021 [55]	USA	Case Series	2	1	1	23.00	2.83	0	2
Marhshall M et al., 2021 [16]	USA	Case Report	1	1	0	18.00	NA	1	0
Matta A et al., 2021 [53]	USA	Case Report	1	1	0	27.00	NA	1	0
Mengesha B et al., 2022 [52]	Israel	Case Report	1	0	1	43.00	NA	1	0
Meyer-Szary J et al., 2022 [51]	Poland	Case Series	1	1	0	29.00	NA	0	1
Miqdad MA et al., 2021 [46]	Saudi Arabia	Case Report	1	1	0	18.00	NA	1	0
Montgomery J et al. 2021 [50]	USA	Retrospective case series	23	23	0	25.00	7.75	7	16
Mouch S et al., 2021 [49]	Israel	Case Series	4	4	0	29.50	10.97	4	0
Murakami Y et al., 2022 [48]	Japan	Case Series	2	2	0	65.00	7.78	2	0
Muthukumar A et al., 2021 [47]	USA	Case Report	1	1	0	52.00	NA	0	1
Nagasaka T et al., 2022 [45]	Japan	Case Report	1	1	0	23.00	NA	1	0
Nevet A et al., 2021 [38]	Israel	Case Series	3	3	0	24.33	4.51	3	0
Nguyen TD et al., 2021 [37]	Germany	Case Report	1	1	0	20.00	NA	0	1
Nunn S et al., 2022 [44]	Germany	Case Series	1	0	1	31.00	NA	1	0
Ohnishi M et al., 2022 [43]	Japan	Case Report	1	1	0	26.00	NA	1	0
Onderko L et al., 2021 [42]	USA	Case Series	3	3	0	29.67	5.69	2	1
Parmar K et al., 2022 [41]	USA	Case Series	4	3	1	29.00	16.06	0	4
Patel YR et al., 2021 [40]	USA	Case Series	5	5	0	24.60	7.30	4	1
Patrignani A et al., 2021 [39]	Italy	Case Report	1	1	0	56.00	NA	1	0
Sano M et al., 2022 [36]	Japan	Case Report	1	1	0	20.00	NA	0	1
Schmitt P et al., 2021 [29]	France	Case Report	1	1	0	19.00	NA	1	0
Shaw KE et al., 2021 [35]	USA	Case Series	2	1	1	27.50	4.95	1	1
Shiyovich A et al., 2022 [34]	Israel	Case Series	4	3	1	27.25	11.64	4	0
Shumkova M et al., 2021 [33]	Bulgaria	Case Report	1	1	0	23.00	NA	1	0
Singh B et al., 2021 [32]	USA	Case Study	1	1	0	24.00	NA	1	0
Sokolska J et al., 2021 [31]	Poland	Case Report	1	1	0	21.00	NA	1	0
Starekova J et al., 2021 [30]	USA	Case Series	4	3	1	27.25	9.36	2	2
Tailor P et al., 2021 [22]	USA	Case Report	1	1	0	44.00	NA	0	1
Verma A et al., 2021 [23]	USA	Case Series	2	1	1	43.50	2.12	1	1
Vidula MK et al., 2021 [24]	USA	Case Report	2	2	0	18.50	0.71	1	1
Viskin D et al., 2021 [21]	Israel	Case Series	8	7	1	NR	NR	8	0
Watkins K et al., 2021 [28]	USA	Case Report	1	1	0	20.00	NA	1	0
Williams CB et al., 2021 [27]	Canada	Case Report	1	1	0	34.00	NA	0	1
Wong J et al., 2022 [26]	Australia	Case Report	1	1	0	20.00	NA	1	0
Wu B et al., 2022 [25]	USA	Case Report	1	1	0	40.00	NA	1	0
Yen KC et al., 2022 [20]	Taiwan	Case Report	1	1	0	32.00	NA	0	1

NA: Not applicable, NR: Not reported, SD: Standard deviation.

**Table 2:** Clinical Characteristics of Included Patients [16,19,20,21,22,23,24,25,26,27,28,29,30,31,32,33,34,35,36,37,38,39,40,41,42,43,44,45,46,47,48,49,50,51,52,53,54,55,56,57,58,59,60,61,62,63,64,65,66,67,68,69,70,71,72,73,74,75,76,77,78,79,80,81,82,83,84,85,86,87,88,89,90,91,92].

Author, Year	Relevant Medical History (n)	Medication History (n)	History of COVID-19 Infection (n)	Symptoms Occurring Following Vaccine Dose (n)			Time to First Symptoms Post-Vaccination (Day)		Symptoms at Time of Admission (n)
				1st Dose	2nd Dose	3rd Dose	Mean	SD	
Abellan C et al., 2021 [92]	None	NR	No (1), NR (2)	0	3	0	2.00	1.00	Chest Pain (3), Chills (2), Emesis (1), Fever (3), Nausea (1), Flu-Like Symptoms (1)
Ahmed SK, 2022 [91]	NR	NR	No (5)	0	5	0	1.80	0.84	Chest Pain (5), SOB (2), Fatigue (3), Fever (2), Headache (1), Myalgia (1)
Aikawa T et al., 2022 [89]	None	NR	NR	0	1	0	2.00	NA	Chest Pain (1)
Alania-Torres E et al., 2021 [90]	ALVC (1), Myocarditis (1), Surgery-ICD Placement (1)	Sacubitril/Valsartan (1), Eplerenone (1), Bisoprolol (1)	No (1)	0	1	0	1.00	NA	Chest Pain (1), SOB (1), Fatigue (1), Fever (1), Headache (1), Myalgia (1), Diarrhoea (1), Muscle Weakness (1)
Albert E et al., 2021 [88]	None	NR	NR	0	1	0	1.00	NA	Chest Pain - Worse on Inspiration and Supine Position (1)
Ammirati E et al., 2021 [87]	None	NR	Yes (1)	0	1	0	3.00	NA	Chest Pain (1)
Aviram G et al., 2022 [86]	Hyperlipidaemia (1), Myocarditis (1), Bilateral Foot Hyperkeratosis (1)	NR	NR	0	0	4	5.75	3.77	Chest Pain (4), Fever (4), Weakness (4)
Bautista Garcia J et al., 2021 [85]	Asthma (1), Atrial Fibrillation (1), Gastritis (1), Hypothyroidism (1), Pneumothorax (1), Surgery - Left Apical Lobectomy (1)	Antipyretics (1), Analgesia (1)	NR	0	1	0	0.25	NA	Chest Pain (1), Fever (1), Interscapular Pain (1)
Bengel C et al., 2022 [84]	ADHD (1)	Methylphenidate (1)	NR	0	2	0	1.00	0.00	Chest Pain (1), Fatigue (1), Fever (1), Malaise (2), Epigastric Pain (1), Arthralgia (1)
Bews H et al., 2022 [83]	IBS (1), Depression (1), Gender Affirming Surgery (2)	Testosterone Therapy (2)	NR	0	9	0	NR	NR	Body Ache (1), Chest Pain (9), Chills (1), Diaphoresis (1), SOB (1), Emesis (1), Fatigue (1), Fever (1), Headache (1), Myalgia (1), Nausea (2), Palpitations (1), Diarrhoea (1), Rhinitis (1), Pharyngitis (1) Pain Worse on Inspiration and Supine Position (1)
Cereda A et al., 2021 [79]	None	NR	No (1)	0	1	0	1.25	NA	Chest Pain (1), Fever (1)
Chachar T et al., 2021 [80]	None	NR	No (1)	1	0	0	5.00	NA	Chest Pain (1)
Chamling B et al., 2021 [81]	Smoker (1)	NR	NR	1	1	0	6.50	5.00	Chest Discomfort (1), Chest Pain (2)
Chellapandian SB et al., 2022 [82]	None	NR	NR	0	1	0	2.00	NA	Body Ache (1), Chest Pain (1)
Chow BT and Lai CK, 2022 [77]	Allergy-Amoxicillin	NR	NR	1	1	0	NR	NR	SOB (1), Fatigue (1), Palpitations (1), Syncope (1), Urinary Incontinence (1), Decreased Exercise Capacity (1), Increased Muscle Tone (1), Foaming at Mouth (1), Tonic-Clonic Movements (1)
Cimaglia P et al., 2022 [78]	None	NSAIDs (1)	NR	0	1	0	2.50	NA	Chest Pain (1), Worse on Inspiration and Supine Position (1)
D’Angelo T et al., 2021 [76]	NR	NR	NR	0	1	0	0.00	NA	Chest Pain (1), Diaphoresis (1), SOB (1), Nausea (1)
Deb A et al., 2021 [75]	Congestive Heart Failure (1), COPD (1), CAD (1), DM (1), GERD (1), HTN (1), Hyperlipidaemia (1), Hypothyroidism (1), Surgery -Multiple Stents, CABG (1)	Aspirin (2), Atorvastatin (1), Clopidogrel (1), Furosemide (1), Isosorbide Mononitrate (1), Levothyroxine (1), Lisinopril (1), Metformin (1), Metoprolol tartrate (1), Potassium Chloride (1), Albuterol and Tiotropium Inhalers (1)	No (1)	0	1	0	0.25	NA	Chills (1), SOB (1), Fatigue (1), Fever (1), Nausea (1), Orthopnoea (1), Coarse Crackles at Lung Bases (1), Elevated JVP (1), Pitting Edema in Lower Extremities (1)
Diaz GA et al., 2021 [74]	CAD (1), DM (2), HTN (5), Alcohol/Drug Dependence (4), Cancer (2), CKD (1)	NR	NR	4	16	0	3.50	5.78	NR
Dickey JB et al., 2021 [73]	None	NR	NR	0	3	0	NR	NR	Chest Pain (3), Chills (2), Fever (1), Myalgia (3), Neck pain (1)
Dlewati M et al., 2022 [72]	Myocarditis (1)	NR	NR	0	1	0	NR	NR	Chest Pain (1), Chills (1), Fatigue (1), Fever (1)
Gautam N et al., 2021 [71]	DM (1), HTN (1), Hyperlipidaemia (1)	NR	NR	0	1	0	90.00	NA	Chest Discomfort (1), Chest Pain (1), Emesis (2)
Habedank D et al., 2022 [70]	HTN (1)	NR	NR	0	1	0	1.00	NA	Fever (1), Palpitations (1), Syncope (1), Dizziness (1)
Habib MB et al., 2021 [69]	NR	Bisoprolol (1)	NR	0	1	0	3.00	NA	Chest Pain (1)
Jahnke C et al., 2022 [68]	None	NR	NR	0	2	0	1.50	NR	Chest Discomfort (2), Chest Pain (2), SOB (2), Malaise (1), Limited physical activity (2)
Kaneta K et al., 2022 [67]	None	None	NR	0	1	0	3.00	NA	Chest Pain (1), Fever (1)
Kaul R et al., 2021 [66]	None	NR	No (2)	0	2	0	NR	NR	Chest Pain (2), Chills (1), Fever (2), Headache (2), Myalgia (1), Neck Pain (1)
Kawakami T et al., 2022 [65]	None	NR	Yes (1)	0	1	0	14.00	NA	Chest Pain (1)
Kim HW et al., 2021 [64]	HTN (1), Hypercholesterolemia (1), Smoker (1)	NR	No (4)	0	4	0	NR	NR	Chest Pain (4), Chills (3), Diaphoresis (1), SOB (3), Fatigue (3), Fever (3), Headache (1), Myalgia (3), Palpitations (1), Syncope (1)
Kim IC et al., 2021 [61]	NR	None	NR	0	1	0	1.00	NA	Chest Pain (1), Fatigue (1), Myalgia (1)
King WW et al., 2021 [63]	NR	NR	No (4)	0	4	0	3.75	1.26	Chest Pain (4)
Koizumi T et al., 2021 [60]	NR	NR	NR	0	2	0	2.50	0.71	Chest Pain (2)
Korosoglou G et al., 2022 [62]	None	NR	NR	0	1	0	2.00	NA	Chest Pain (1), Fatigue (1), Headache (1), Myalgia (1), Joint Pain (1), - Worse on Inspiration (1)
Larson KF et al., 2021 [59]	NR	NR	Yes (2)	1	7	0	1.17	0.41	Chest Pain (3), Chills (2), Cough (1), Fever (2)
Lee CH and Kong EJ, 2022 [58]	None	NR	NR	0	1	0	5.00	NA	Chest Pain (1)
Levin D et al., 2021 [56]	Asthma (1), Myocarditis (1), ADHD (1), Celiac Disease (1)	NR	NR	2	5	0	NR	NR	Chest Pain (6), Fatigue (6), Fever (2), Headache (2), Abdominal Pain (1)
Maeda M et al., 2022 [19]	None	NR	No (1)	1	0	0	4.00	NA	Chest Pain (1)
Maki H et al., 2022 [54]	Kawasaki Disease (1)	NR	NR	0	1	0	14.00	NA	Chest Pain (1), SOB (1), Fever (1)
Manfredi R et al., 2022 [57]	RBBB (1)	None	NR	0	2	0	NR	NR	Fever (2)
Mansour J et al., 2021 [55]	None	NR	No (2)	0	2	0	1.00	0.00	Chest Pain (2), Chills (1), Fever (1), Lightheaded (1)
Marhshall M et al., 2021 [16]	None	NR	No (1)	0	1	0	3.00	NA	Chest Pain (1), Fever (1), Malaise (1), Myalgia (1), Arthralgia (1)
Matta A et al., 2021 [53]	NR	NR	No (1)	0	1	0	3.00	NA	Chest Pain (1), Fatigue (1)
Mengesha B et al., 2022 [52]	Obesity (1)	None	No (1)	0	0	1	2.00	NA	SOB (1), Palpitations (1)
Meyer-Szary J et al., 2022 [51]	NR	NR	No (1)	0	1	0	2.00	NA	Chest Discomfort (1), Chest Pain (1), SOB (1), Pain Radiates To Left Arm (1)
Miqdad MA et al., 2021 [46]	None	NR	NR	0	1	0	4.00	NA	Chest Pain - Worse on Inspiration (1)
Montgomery J et al. 2021 [50]	NR	NR	No (2), Yes (3)	3	20	0	2.08	0.88	Chest Pain (23)
Mouch S et al., 2021 [49]	Hyperlipidaemia (1), Obesity (1), Smoker (1)	NR	NR	1	3	0	5.75	6.85	Chest Discomfort (1), Chest Pain (3), Pain Worse on Inspiration (1)
Murakami Y et al., 2022 [48]	NR	NR	NR	1	1	0	6.50	3.54	Chest Pain (2)
Muthukumar A et al., 2021 [47]	HTN (1), Hypercholesterolemia (1), OSA (1), LFT Elevations (1)	Aspirin (1), Simvastatin (1), Ezetimibe (1), Lisinopril (1)	No (1)	0	1	0	3.00	NA	Chest Pain (1), Headache (1)
Nagasaka T et al., 2022 [45]	None	NR	No (1)	0	1	0	3.00	NA	Chest Pain (1), Fever (1)
Nevet A et al., 2021 [38]	NR	NR	NR	0	3	0	2.00	NR	Chest Pain (3), Fever (3)
Nguyen TD et al., 2021 [37]	None	NR	Yes (1)	1	0	0	0.50	NA	Chest Pain (1), Fatigue (1), Fever (1), Myalgia (1), Shivering (1)
Nunn S et al., 2022 [44]	None	NR	NR	1	0	0	NR	NR	Chest Pain (1), SOB (1), Shivering (1)
Ohnishi M et al., 2022 [43]	None	NR	NR	0	1	0	2.00	NA	Chest Pain (1), Fever (1), Headache (1), Loss of Appetite (1)
Onderko L et al., 2021 [42]	GERD (1), Crohn’s Disease (1), Obesity (1)	None	NR	0	3	0	NR	NR	Chest Pain (3), Jaw pain (1), Pain Worse on Inspiration (1)
Parmar K et al., 2022 [41]	Asthma (2), Hyperlipidaemia (1), Depression (1), Kidney Stones (1), Fibrous Dysplasia (1)	NR	NR	1	3	0	NR	NR	Chest Pain (3), Pain In Left Arm (1)
Patel YR et al., 2021 [40]	Asthma (1), ADHD (1)	NR	No (5)	1	4	0	2.20	0.84	Body Ache (2), Chest Pain (5), Chills (1), Diaphoresis (1), SOB (3), Emesis (1), Fatigue (2), Fever (1), Headache (4), Malaise (1), Myalgia (1), Nausea (3), Rigors (1), Worse on Inspiration and Supine Position (1)
Patrignani A et al., 2021 [39]	None	NR	Yes (1)	1	0	0	4.00	NA	Diaphoresis (1), Epigastric Pain (1)
Sano M et al., 2022 [36]	None	NR	NR	0	1	0	2.00	NA	Chest Pain (1), Fever (1)
Schmitt P et al., 2021 [29]	None	NR	NR	0	1	0	3.00	NA	Chest Pain (1), Worse on Inspiration and Supine Position (1)
Shaw KE et al., 2021 [35]	NR	NR	No (1), Yes (1)	1	1	0	14.50	14.85	Chest Discomfort (1), Chest Pain (1)
Shiyovich A et al., 2022 [34]	Asthma (1)	NR	NR	0	0	4	5.75	4.80	Chest Pain (4)
Shumkova M et al., 2021 [33]	None	NR	No (1)	0	1	0	1.00	NA	Chest Pain (1), SOB (1), Fever (1), Worse on Inspiration and Supine Position (1)
Singh B et al., 2021 [32]	None	None	No (1)	0	1	0	3.00	NA	Chest Pain (1), Headache (1)
Sokolska J et al., 2021 [31]	Asthma (1), Surgery-Appendectomy (1), Allergy - Pollen, Pets (1)	Analgesia (1)	No (1)	1	0	0	3.00	NA	Chest Discomfort (1), Chest Pain (1), Injection Site Discomfort (1)
Starekova J et al., 2021 [30]	NR	NR	No (4)	0	4	0	NR	NR	Body Ache (1), Chest Discomfort (2), Chest Pain (4), Chills (2), SOB (2), Fatigue (1), Fever (2), Headache (2), Malaise (2), Myalgia (1), Nausea (1), Lightheaded (1)
Tailor P et al., 2021 [22]	Asthma (1), OSA (1), Obesity (1), Previous Smoker (1)	Asthma Medication - Albuterol, Fluticasone-Salmeterol (2)	No (1)	0	1	0	4.00	NA	Chest Pain (1), Cough (1), SOB (1), Headache (1), Malaise (1), Myalgia (1)
Verma A et al., 2021 [23]	Overweight (1), Smoker (1)	None	NR	1	1	0	NR	NR	Chest Pain (1), SOB (2), Dizziness (1)
Vidula MK et al., 2021 [24]	None	NR	NR	0	2	0	2.50	2.12	Chest Discomfort (1), Chest Pain (1), SOB (1), Fever (1), Myalgia (1)
Viskin D et al., 2021 [21]	Asthma (1), Celiac Disease (1), Peri myocarditis (1)	Asthma Medication - Montelukast therapy (1)	No (1)	0	8	0	NR	NR	Chest Pain (8), Malaise (8)
Watkins K et al., 2021 [28]	Smoker (1)	NR	Yes (1)	0	1	0	2.00	NA	Chest Pain (1), SOB (1), Worse with sitting compared to supine (1)
Williams CB et al., 2021 [27]	NR	None	NR	0	1	0	1.00	NA	Chest Pain (1), Fever (1), Myalgia (1), Tachycardia (1), Tachypnea (1), Elevated JVP (1), Mild Crackles At Lung Bases (1)
Wong J et al., 2022 [26]	Asthma (1), Depression (1)	Asthma Medication - Fluticasone/Salmeterol (1), Mirtazapine (1)	NR	0	1	0	0.50	NA	Chest Pain (1), Chills (1), Diaphoresis (1), Fever (1), Headache (1), Night Sweats (1)
Wu B et al., 2022 [25]	None	Testosterone Therapy (1)	Yes (1)	1	0	0	NR	NR	Chest Pain (1), SOB (1), Fever (1), Myalgia (1), Nausea (1)
Yen KC et al., 2022 [20]	Hyperlipidaemia (1), Gouty Arthritis (1)	NR	NR	1	0	0	7.00	NA	Chest Discomfort (1), SOB (1), Injection Site Discomfort (1), Loose Stools (1)

ALVC: Arrhythmogenic left ventricular cardiomyopathy, ADHD: Attention deficit hyperactivity disorder, CABG: Coronary artery bypass graft, CAD: coronary artery disease, COPD: Chronic obstructive pulmonary disorder, GERD: Gastric esophageal reflux disease, HTN: Hypertension, ICD: Implantable cardioverter defibrillator, NA: Not applicable, NR: Not reported, OSA: Obstructive sleep apnea, SD: Standard deviation, SOB: shortness of breath.

**Table 3:** Major Medical Imaging Findings on Admission.

Author, Year	Imaging Conducted (n)	Echo Findings (n)	CT Angiography Findings (n)	Coronary Angiogram Findings (n)	ECG Findings (n)	MRI Findings (n)	Myocarditis Diagnostic Tool
Abellan C et al., 2021 [92]	Echocardiogram (3), Coronary Angiogram (2), ECG (3), MRI (3)	Hypokinesia (2), Reduced LVEF (1), Unremarkable (1)	NR	Unremarkable (2)	ST Elevation (2), PR Depression (2), Incomplete RBBB (1), ST Depression (1), Inverted T Wave (1)	Subepicardial Enhancement (3), Myocardial Edema (2), Pericardial Effusion (1)	Cardiac MRI (3)
Ahmed SK, 2022 [91]	CXR (5), Echocardiogram (5), ECG (5),	NR	NR	NR	ST Changes (5)	NR	Troponin Levels and Echocardiogram (5)
Aikawa T et al., 2022 [89]	Echocardiogram (1), CT Angiography (1), Cardiac Biopsy (1), ECG (1), MRI (1)	Wall Motion Abnormalities (1)	Subepicardial Delayed Enhancement (1)	NR	ST Elevation (1)	Subepicardial Enhancement (1)	Cardiac MRI (1)
Alania-Torres E et al., 2021 [90]	CXR (1), Echocardiogram (1), ECG (1),	LV Enlargement (1), LV Systolic Dysfunction (1)	NR	NR	QRS Changes (1), Flat T Waves (1)	NR	Laboratory Data, Clinical Symptoms (1)
Albert E et al., 2021 [88]	Echocardiogram (1), CT Angiography (1), ECG (1), MRI (1)	Unremarkable (1)	Bilateral Pleural Effusion (1)	NR	Unremarkable (1)	Myocardial Enhancement (1), Epicardial Enhancement (1), Edema (1)	Lake Louise Criteria (1)
Ammirati E et al., 2021 [87]	CXR (1), CT Angiography (1), Cardiac Ventriculography (1), Coronary Angiogram (1), ECG (1), MRI (1)	NR	Unremarkable (1)	Unremarkable (1)	ST Elevation (1), Peaked T Wave (1)	Unremarkable (1)	Cardiac MRI (1)
Aviram G et al., 2022 [86]	CXR (4), Echocardiogram (4), Chest CT (4), ECG (4), MRI (4)	Unremarkable (2), Reduced LVEF (2)	NR	NR	ST Elevation (2), Unremarkable (2)	Edema (1), Subepicardial Enhancement (2)	Cardiac MRI (4)
Bautista Garcia J et al., 2021 [85]	CXR (1), Echocardiogram (1), CT Angiography (2), ECG (2), MRI (1)	Unremarkable (1)	Unremarkable (2)	NR	Sinus Tachycardia (1), Narrow QRS Complex (1), ST Elevation (1)	Edema (1), Subepicardial Enhancement (1)	Cardiac MRI (1)
Bengel C et al., 2022 [84]	CXR (1), Echocardiogram (4), Coronary Angiogram (1), ECG (3), MRI (2)	Unremarkable (1), Reduced Systolic Function (1), Hypokinesis (1)	NR	Unremarkable (1)	ST Elevation (1), ST Depression (1)	Myocardial Edema (2), Hypokinesis (1), Reduced LV Systolic Function (1), Myocardial Enhancement (1), Pericardial Enhancement (1)	Lake Louise Criteria (1), Cardiac MRI (1)
Bews H et al., 2022 [83]	Echocardiogram (9), ECG (9), MRI (9)	Wall Motion Abnormalities (6)	NR	NR	ST Elevation (1), PR Depression (1)	Subepicardial Enhancement (7), Myocardial Enhancement (5), Hypokinesis (1)	Cardiac MRI (9)
Cereda A et al., 2021 [79]	CXR (1), Echocardiogram (1), Coronary Angiogram (1), ECG (1), MRI (1)	Hypokinesis (1)	NR	Unremarkable (1)	ST Elevation (1)	Edema (1), Epicardial Enhancement (1)	Cardiac MRI (1)
Chachar T et al., 2021 [80]	Echocardiogram (1), Coronary Angiogram (1), ECG (1), MRI (1)	Unremarkable (1)	NR	Unremarkable (1)	ST Elevation (1)	Subepicardial Enhancement (1)	Cardiac MRI (1)
Chamling B et al., 2021 [81]	Echocardiogram (2), Cardiac Ventriculography (1), Coronary Angiogram (1), Heart Catheterisation (1), ECG (2), MRI (2)	Unremarkable (2)	NR	Unremarkable (1)	ST Elevation (2)	Inflammatory Focus (1), Non-Ischemic Myocardial Damage (1)	Cardiac MRI (2)
Chellapandian SB et al., 2022 [82]	CXR (1), Echocardiogram (1), CT Angiography (1), ECG (1), MRI (1)	Unremarkable (1)	Unremarkable (1)	NR	Unremarkable (1)	Myocardial Edema (1), Hyperaemia (1), Scarring (1)	Cardiac MRI (1)
Chow BT and Lai CK, 2022 [77]	Echocardiogram (1), Coronary Angiogram (1), Heart Catheterisation (1), ECG (2), MRI (1)	Reduced LVEF (1), Wall Motion Abnormalities (1), Mitral Regurgitation (1)	NR	Coronary Artery Disease (1)	T Wave Inversion (1), QRS Changes (1)	Hypokinesia (1), Subepicardial Enhancement (1), Reduced RV Systolic Function (1)	Lake Louise Criteria (1)
Cimaglia P et al., 2022 [78]	Echocardiogram (1), Coronary Angiogram (1), ECG (1), MRI (1)	Reduced LVEF (1), Hypokinesia (1), Pericardium Hyper Echogenicity (1)	NR	Unremarkable (1)	ST Elevation (1), ST Depression (1)	Dilated Left Ventricle (1), Myocardial Enhancement (1)	Lake Louise Criteria (1)
D’Angelo T et al., 2021 [76]	Echocardiogram (1), Coronary Angiogram (1), ECG (1), MRI (1)	Pericardial Effusion (1), Hypokinesia (1)	NR	Unremarkable (1)	ST Elevation (1), Nonspecific T Wave Change (1)	Myocardial Enhancement (1), Pericardial Enhancement (1)	ECG, Cardiac MRI, Laboratory Data (1)
Deb A et al., 2021 [75]	CXR (1), Echocardiogram (1), ECG (1)	Dilated Left Atrium (1), Hypokinesia (1), Grade 2 Diastolic Dysfunction (1)	NR	NR	Sinus Tachycardia (1), Nonspecific ST Change (1), Nonspecific T Wave Change (1)	NR	Cardiac MRI (1)
Diaz GA et al., 2021 [74]	ECG (NR), MRI (20)	NR	NR	NR	Bundle Branch Block (1), ST Elevation (9)	NR	MRI + Lab Findings (20)
Dickey JB et al., 2021 [73]	ECG (3), MRI (3)	NR	NR	NR	ST Elevation (3), PR Depression (1)	Myocardial Enhancement (1)	Cardiac MRI (3)
Dlewati M et al., 2022 [72]	Echocardiogram (1), Cardiac Ventriculography (1), Coronary Angiogram (1), Heart Catheterisation (1), ECG (1)	Unremarkable (1)	NR	Unremarkable (1)	ST Elevation (1), ST Depression (1)	NR	Clinical Findings (1)
Gautam N et al., 2021 [71]	CXR (1), Coronary Angiogram (1), ECG (1), MRI (1)	NR	NR	Non-Obstructive Coronary Artery Disease (1)	ST Elevation (1)	Reduced LVEF (1), Myocardial Enhancement (1), Epicardial Enhancement (1)	Cardiac MRI (1)
Habedank D et al., 2022 [70]	Echocardiogram (1), Coronary Angiogram (1), ECG (1), MRI (1)	Unremarkable (1)	NR	Unremarkable (1)	Unremarkable (1)	Focal Edema (1), Subepicardial Enhancement (1)	Cardiac MRI (1)
Habib MB et al., 2021 [69]	Echocardiogram (1), CT Angiography (1), ECG (1), MRI (1)	Unremarkable (1)	Unremarkable (1)	NR	ST Elevation (1)	Subepicardial Enhancement (1)	Cardiac MRI (1)
Jahnke C et al., 2022 [68]	Echocardiogram (2), Coronary Angiogram (2), ECG (2), MRI (2)	Unremarkable (2)	NR	Unremarkable (2)	Unremarkable (2)	Regional Wall Motion Abnormality (1), Pericardial Effusion (1), Unspecified Enhancement (1)	Cardiac MRI (2)
Kaneta K et al., 2022 [67]	Echocardiogram (1), Cardiac Ventriculography (1), Coronary Angiogram (1), MRI (1)	Hypokinesia (1)	NR	Unremarkable (1)	ST Elevation (1)	Unspecified Enhancement (1)	Lake Louise Criteria (1)
Kaul R et al., 2021 [66]	Echocardiogram (4), Coronary Angiogram (1), ECG (2), MRI (2)	Hypokinesis (1), Dilated RV (1), Reduced Systolic Function (1), Mitral Regurgitation (1), Unremarkable (2)	NR	Unremarkable (1)	ST Elevation (2), ST Depression (1)	Delayed Epicardium Enhancement (1), Delayed Pericardium Enhancement (1)	Lake Louise Criteria (2)
Kawakami T et al., 2022 [65]	CXR (1), Echocardiogram (1), Cardiac Ventriculography (1), Coronary Angiogram (1), ECG (1), MRI (1)	Reduced LVEF (1), Wall Motion Abnormalities (1)	NR	Unremarkable (1)	T Wave Inversion (1)	Myocardial Edema (1)	Lake Louise Criteria (1)
Kim HW et al., 2021 [64]	CXR (4), Chest CT (2), Coronary Angiogram (1), ECG (4), MRI (4)	NR	NR	Unremarkable (1)	ST Elevation (2), PR Depression (2)	Reduced LVEF (1), Pericardial Effusion (4), Regional Wall Motion Abnormality (4)	Cardiac MRI (4)
Kim IC et al., 2021 [61]	Echocardiogram (1), CT Angiography (1), ECG (1), MRI (1)	Worsened Strain Value (1)	Unremarkable (1)	NR	ST Elevation (1)	Unspecified Increased Signal Intensity (1), Subepicardial Enhancement (1)	Lake Louise Criteria (1)
King WW et al., 2021 [63]	Echocardiogram (4), ECG (4), MRI (1)	Hypokinesis (2)	NR	NR	PR Depression (3), ST Elevation (3), T Wave Inversion (1)	Subepicardial Enhancement (1)	Cardiac MRI (1), TTE, ECG (3)
Koizumi T et al., 2021 [60]	Echocardiogram (2), Coronary Angiogram (2), Cardiac Biopsy (2), ECG (2), MRI (1)	Unremarkable (2)	NR	Unremarkable (2)	ST Elevation (2)	Epicardial Enhancement (1)	Cardiac MRI (1), ECG, Laboratory Data (1)
Korosoglou G et al., 2022 [62]	Echocardiogram (1), ECG (1), MRI (1)	Reduced LVEF (1)	NR	NR	ST Elevation (1)	Reduced LVEF (1), Myocardial Edema (1), Subepicardial Enhancement (1), Pericardial Effusion (1)	Cardiac MRI (1)
Larson KF et al., 2021 [59]	Heart Catheterisation (5), Cardiac Biopsy (1), ECG (8), MRI (8)	NR	NR	NR	ST Elevation (6), Peaked T Wave (1)	Subepicardial Enhancement (1), Myocardial Enhancement (1), Edema (6), Delayed Enhancement (6), Pericardial Effusion (3), Pericardial Edema (1)	Cardiac MRI (8)
Lee CH and Kong EJ, 2022 [58]	Echocardiogram (1), ECG (1), MRI (1)	Unremarkable (1)	NR	NR	ST Depression (1), T Wave Inversion (1)	Subepicardial Enhancement (1)	F-FDG PET/MRI (1)
Levin D et al., 2021 [56]	Echocardiogram (7), CT Angiography (2), Heart Catheterisation (1), ECG (7), MRI (3)	Hypokinesis (1), LV Dysfunction (1), Reduce LVEF (2), Unremarkable (5)	Subepicardial Focal Enhancement (1), Late Wall Adherence (1)	NR	ST Elevation (6), PR Depression (2), Sinus Tachycardia (1)	Subepicardial Enhancement (3), Mesocardiac Enhancement (1), Myocardial Edema (1), Dilated LV (1), Systolic Dysfunction (1), Myocardium Enhancement (1)	ECG (2), CT Scan (2), Cardiac MRI (3)
Maeda M et al., 2022 [19]	Echocardiogram (1), Coronary Angiogram (1), ECG (1), MRI (1)	Unremarkable (1)	NR	Unremarkable (1)	Nonspecific ST Changes (1), Nonspecific T Wave Changes (1)	Myocardial Edema (1)	Cardiac MRI (1)
Maki H et al., 2022 [54]	CXR (1), Echocardiogram (1), Coronary Angiogram (1), Cardiac Biopsy (1), ECG (1), MRI (1)	Hypokinesis (1), Pericardial Effusion (1)	NR	Unremarkable (1)	Sinus Tachycardia (1), ST Elevation (1)	Biventricular Systolic Dysfunction (1)	EMB (1)
Manfredi R et al., 2022 [57]	Echocardiogram (2), ECG (2), MRI (2)	Unremarkable (2)	NR	NR	Unremarkable (2)	Myocardial Edema (1) Unspecified Enhancement (1)	Cardiac MRI (2)
Mansour J et al., 2021 [55]	Echocardiogram (2), CT Angiography (1), Coronary Angiogram (1), ECG (2), MRI (2)	Reduced LVEF (1)	Unremarkable (1)	Unremarkable (1)	ST Elevation (2), PR Depression (1)	Subepicardial Enhancement (2), Unspecified Increased Signal Intensity (2)	Cardiac MRI, Lake Louise criteria (2)
Marhshall M et al., 2021 [16]	Echocardiogram (1), ECG (1), MRI (1)	Unremarkable (1)	NR	NR	ST elevation (1)	Myocardial Edema (1), Hyperaemia (1), Fibrosis (1), Mitral Regurgitation (1)	Cardiac MRI (1)
Matta A et al., 2021 [53]	CXR (1), Echocardiogram (1), ECG (1),	Unremarkable (1)	NR	NR	Unremarkable (1)	NR	Laboratory Data, Clinical Symptoms (1)
Mengesha B et al., 2022 [52]	Echocardiogram (1), CT Angiography (1), Coronary Angiogram (1), Heart Catheterisation (1), Cardiac Biopsy (1), ECG (1), MRI (1)	Reduced LVEF (1), Enlarged Left Ventricle (1), Mitral Regurgitation (1), Tricuspid Regurgitation (1)	Unremarkable (1)	Unremarkable (1)	Sinus Tachycardia (1)	Reduced Systolic Function (1), Hypokinesia (1), Akinesia (1), Myocardial Edema (1), Subendocardial Enhancement (1)	EMB (1)
Meyer-Szary J et al., 2022 [51]	Echocardiogram (1), Chest CT (1), Coronary Angiogram (1), ECG (1), MRI (1)	Unremarkable (1)	NR	Unremarkable (1)	NR	Edema (1), Myocardial Injury (1), Delayed Subepicardial Enhancement (1)	Cardiac MRI (1)
Miqdad MA et al., 2021 [46]	Echocardiogram (1), Coronary Angiogram (1), ECG (1), MRI (1)	Unremarkable (1)	NR	Unremarkable (1)	ST Elevation (1)	Myocardial Edema (1)	Cardiac MRI (1)
Montgomery J et al. 2021 [50]	Echocardiogram (23), CT Angiography (5), Coronary Angiogram (11), Heart Catheterisation (11), ECG (23), MRI (8)	Reduced LVEF (4)	Unremarkable (5)	Unremarkable (11)	NR	Subepicardial Enhancement (8), Myocardial Edema (8)	Lake Louise Criteria (8), Diagnosis Not Performed (15)
Mouch S et al., 2021 [49]	Echocardiogram (4), CT Angiography (1), Coronary Angiogram (1), ECG (4), MRI (4)	Reduced LVEF (1), Hypokinesis (1)	Unremarkable (1)	Unremarkable (1)	Sinus Tachycardia (1), ST Elevation (4), ST Depression (1), T Wave Inversion (3), PR Depression (2)	Myocardial Edema (4), Subepicardial Edema (3), Myocardial Enhancement (4), Subepicardial Enhancement (5)	Cardiac MRI (4)
Murakami Y et al., 2022 [48]	Echocardiogram (2), CT Angiography (1), Coronary Angiogram (1), ECG (2), MRI (2)	Unremarkable (1), Hypokinesis (1)	Unremarkable (1)	Unremarkable (1)	ST Elevation (1)	Subepicardial Enhancement (1), Myocardial Edema (1), Subepicardial Lesion (1)	Cardiac MRI (2)
Muthukumar A et al., 2021 [47]	Echocardiogram (1), Coronary Angiogram (1), ECG (1), MRI (1)	Unremarkable (1)	NR	Non-Obstructive Coronary Artery Disease (1)	Incomplete Right BBB (1)	Myocardial Enhancement (1), Subepicardial Enhancement (1)	Lake Louise Criteria (1)
Nagasaka T et al., 2022 [45]	CXR (1), Echocardiogram (1), Coronary Angiogram (1), Cardiac Biopsy (1), ECG (1), MRI (1)	Wall Motion Abnormality (1), Pericardial Effusion (1)	NR	Unremarkable (1)	ST Elevation (1)	Subepicardial Enhancement (1), Myocardial Enhancement (1)	EMB and Cardiac MRI (1)
Nevet A et al., 2021 [38]	Echocardiogram (3), ECG (3), MRI (3)	Unremarkable (3)	NR	NR	ST Elevation (3)	Myocardial Edema (1), Myocardial Enhancement (1)	Cardiac MRI (3)
Nguyen TD et al., 2021 [37]	CXR (1), Echocardiogram (1), Cardiac Biopsy (1), ECG (1), MRI (1)	Unremarkable (1)	NR	NR	PR Changes (1), Delta Waves (1), T Wave Changes (1)	Subepicardial Enhancement (1), Myocardial Edema (1)	Cardiac MRI (1)
Nunn S et al., 2022 [44]	Echocardiogram (1), Heart Catheterisation (1), Cardiac Biopsy (1), MRI (1)	Wall Motion Abnormality (1)	NR	NR	NR	Subepicardial Scarring (1), Subepicardial Enhancement (1)	Left Heart Catheterisation and EMB (1)
Ohnishi M et al., 2022 [43]	CXR (1), Echocardiogram (1), Chest CT (1), Coronary Angiogram (1), ECG (1), MRI (1)	Unremarkable (1)	NR	Coronary Artery Fistula (1)	Q Wave Change (1), ST Elevation (1), ST Depression (1)	Subepicardial Enhancement (1)	Cardiac MRI (1)
Onderko L et al., 2021 [42]	Echocardiogram (2), Coronary Angiogram (2), ECG (3), MRI (3)	Unremarkable (1)	NR	Unremarkable (2)	Unremarkable (1), STEMI (2)	Myocardial Edema (3), Myocardial Injury (3)	Cardiac MRI (3)
Parmar K et al., 2022 [41]	CXR (4), Echocardiogram (4), Heart Catheterisation (2), Cardiac Biopsy (1), ECG (4), MRI (4)	NR	NR	NR	AV Block (1), Unremarkable (1), ST Elevation (2)	Delayed Pericardial Enhancement (1), Unspecified Enhancement (2)	EMB (1), Cardiac MRI (2), European Society of Cardiology 2013 Criteria (1)
Patel YR et al., 2021 [40]	Echocardiogram (5), CT Angiography (1), Coronary Angiogram (2), Heart Catheterisation (1), ECG (5), MRI (5)	Unremarkable (5)	Unremarkable (1)	Unremarkable (2)	Sinus Tachycardia (1), PR Depression (3), PR Elevation (3), ST Elevation (1), ST Depression (1)	Subepicardial Enhancement (4), Myocardial Enhancement (1), Myocardial Edema (2)	Lake Louise Criteria (5)
Patrignani A et al., 2021 [39]	CXR (1), Echocardiogram (1), Coronary Angiogram (1), ECG (1), MRI (1)	Unremarkable (1)	NR	Unremarkable (1)	Unremarkable (1)	Myocardial Edema (1), Subepicardial Enhancement (1)	Cardiac MRI (1)
Sano M et al., 2022 [36]	CXR (1), Echocardiogram (1), CT Angiography (1), Chest CT (1), Coronary Angiogram (1), Heart Catheterisation (1), Cardiac Biopsy (1), ECG (1), MRI (1)	Reduced LVEF (1), Hypokinesis (1)	Unremarkable (1)	Unremarkable (1)	ST Elevation (1), PR Depression (1)	Reduced LVEF (1), Hyperaemia (1), Myocardial Edema (1), Subepicardial Enhancement (1), Myocardial Enhancement (1),	EMB and Cardiac MRI (1)
Schmitt P et al., 2021 [29]	Echocardiogram (1), ECG (1), MRI (1)	Unremarkable (1)	NR	NR	ST Elevation (1)	Subepicardial Enhancement (1), Myocardial Edema (1),	Cardiac MRI and Lake Louise criteria (1)
Shaw KE et al., 2021 [35]	MRI (2)	NR	NR	NR	NR	Unremarkable (2)	Lake Louise Criteria (2)
Shiyovich A et al., 2022 [34]	CT Angiography (2), Coronary Angiogram (1), ECG (4), MRI (4)	NR	Unremarkable (2)	Unremarkable (1)	ST Elevation (3), Unremarkable (1)	Regional Wall Motion Abnormalities (1), Unspecified Enhancement (4)	Lake Louise Criteria (4)
Shumkova M et al., 2021 [33]	Echocardiogram (1), Cardiac Ventriculography (1), Coronary Angiogram (1), ECG (1), MRI (1)	Reduced LVEF (1), Hypokinesis (1)	NR	Unremarkable (1)	ST Elevation (1)	Reduced LVEF (1)	Lake Louise Criteria (1)
Singh B et al., 2021 [32]	CXR (1), Echocardiogram (1), CT Angiography (1), Coronary Angiogram (1), Heart Catheterisation (1), ECG (1), MRI (1)	Unremarkable (1)	Unremarkable (1)	Unremarkable (1)	ST Depression (1)	Subepicardial Enhancement (1)	Cardiac MRI (1)
Sokolska J et al., 2021 [31]	Echocardiogram (1), CT Angiography (1), ECG (1), MRI (1)	Global Longitudinal Deformation (1)	Unremarkable (1)	NR	Incomplete RBBB (1), Q Wave Elevation (1), ST Elevation (1), Negative T Wave (1)	Unspecified Increased Signal Intensity (1), Subepicardial Enhancement (1)	Cardiac MRI (1)
Starekova J et al., 2021 [30]	CT Angiography (3), Pulmonary MRA (2), Scintigraphy (1), ECG (4), MRI (4)	NR	Unremarkable (3)	NR	ST Elevation (1), ST Depression (1), Nonspecific T Wave Change (2), T Wave Inversion (1)	Myocardial Enhancement (2), Epicardial Enhancement (5), Pericardial Enhancement (5), Pericardial Effusion (2), Ipsilateral Axillary Lymphadenopathy (4)	Lake Louise Criteria (4)
Tailor P et al., 2021 [22]	CXR (1), Echocardiogram (1), Cardiac Ventriculography (1), Coronary Angiogram (1), Heart Catheterisation (1), ECG (1), MRI (1)	Enlarged Left Ventricle (1), Enlarged Right Ventricle (1), Reduced LVEF (1), Hypokinesis (1)	NR	Coronary Artery Disease (1)	ST Elevation (1)	Myocardial Enhancement (1) Subepicardial Enhancement (1)	Cardiac MRI (1)
Verma A et al., 2021 [23]	Echocardiogram (2), Coronary Angiogram (2), Heart Catheterisation (1), Cardiac Biopsy (1), ECG (2), MRI (1)	Hypokinesis (2), Reduced LVEF (2), Grade 1 Diastolic Dysfunction (2), Pericardial Effusion (2), RV Systolic Dysfunction (1), LV Hypertrophy (1), Dilated IVC (1)	NR	Unremarkable (2)	Sinus Tachycardia (1), ST Depression (1), ST Elevation (1)	Subepicardial Enhancement (1), Pericardial Enhancement (1)	NR (1), Autopsy (1)
Vidula MK et al., 2021 [24]	Echocardiogram (2), CT Angiography (1), Coronary Angiogram (1), ECG (2), MRI (2)	Reduced LVEF (2)	Unremarkable (1)	Unremarkable (1)	Sinus Tachycardia (1), ST Elevation (2)	Hypokinesis (2), Subepicardial Enhancement (2)	Lake Louise Criteria (2)
Viskin D et al., 2021 [21]	CXR (8), Echocardiogram (8), CT Angiography (4), ECG (5), MRI (7)	Reduced LVEF (3), Impaired Speckled Strain (5)	Typical Myocarditis Distribution (4)	NR	Nonspecific ST Change (5), Nonspecific Q Wave Change (1)	Subepicardial Enhancement (6), Myocardial Enhancement (6), Myocardial Edema (1)	Cardiac MRI (6), Clinical Symptoms, ECG, Laboratory Data, Echocardiography (2)
Watkins K et al., 2021 [28]	Ultrasound (1), Echocardiogram (1), CT Angiography (1), Coronary Angiogram (1), Heart Catheterisation (1), ECG (1), MRI (1)	Unremarkable (1)	Unremarkable (1)	Unremarkable (1)	Sinus Tachycardia (1), ST Elevation (1), PR Depression (1)	Stated Positive for Myocarditis (1)	Cardiac MRI (1)
Williams CB et al., 2021 [27]	CXR (1), Echocardiogram (1), ECG (1), MRI (1)	Reduced LVEF (1)	NR	NR	Sinus Tachycardia (1), PR Depression (1), ST Elevation (1), PR Elevation (1), ST Depression (1)	Subepicardial Enhancement (1), Myocardial Edema (1)	Lake Louise Criteria (1)
Wong J et al., 2022 [26]	CXR (1), Echocardiogram (1), ECG (1), MRI (1)	Unremarkable (1)	NR	NR	Sinus Tachycardia (1), ST Elevation (1)	Reduced Systolic Function (1), Hypokinesis (1), Delayed Epicardial Enhancement (1)	Cardiac MRI (1)
Wu B et al., 2022 [25]	CXR (1), Echocardiogram (1), Coronary Angiogram (1), Heart Catheterisation (1), Cardiac Biopsy (1), ECG (1), MRI (1)	Hypokinesis (1), Reduced LVEF (1), Pericardial Effusion (1)	NR	Unremarkable (1)	ST Elevation (1), ST Depression (1)	Reduced LVEF (1), Myocardial Edema (1), Delayed Myocardial Enhancement (1)	Cardiac MRI and EMB (1)
Yen KC et al., 2022 [20]	Echocardiogram (1), Coronary Angiogram (1), ECG (1), MRI (1)	Unremarkable (1)	NR	Unremarkable (1)	Sinus Tachycardia (1)	Subepicardial Enhancement (1), Focal Edema (1)	Cardiac MRI (1)

Current guidelines for Lake Louise Criteria state a diagnosis of myocarditis if cardiac MRI demonstrates myocardial edema from T2 mapping/T2 weighted imaging and non-ischemic myocardial injury from abnormal T1 mapping/extracellular volume/late gadolinium enhancement. Pericarditis and systolic LV dysfunction are considered supportive criteria. BBB: Bundle branch block, Cardiac MRI: Cardiac magnetic resonance imaging, CT: Computerized tomography, CXR: Chest X-ray, ECG: Electrocardiogram, EMB: Endomyocardial biopsy, IVC: Inferior vena cava, LV: Left ventricle, LVEF: Left ventricular ejection fraction, MRA: Magnetic resonance angiography, NR: Not reported, RV: Right ventricle, TTE: Transthoracic echocardiogram.

**Table 4:** Major Laboratory Findings on Admission [16,19,20,21,22,23,24,25,26,27,28,29,30,31,32,33,34,35,36,37,38,39,40,41,42,43,44,45,46,47,48,49,50,51,52,53,54,55,56,57,58,59,60,61,62,63,64,65,66,67,68,69,70,71,72,73,74,75,76,77,78,79,80,81,82,83,84,85,86,87,88,89,90,91,92].

Author, Year	ESR (mm/hr)		CRP (mg/L)		cTnI (ng/mL)		Elevated Troponin (n)	PCR for SARS-CoV2	Viral and Bacterial Infection Serology
	mean	SD	mean	SD	mean	SD			
Abellan C et al., 2021 [92]	NR	NR	54.67	34.77	NR	NR	3	Negative (3)	NR
Ahmed SK, 2022 [91]	NR	NR	NR	NR	NR	NR	5	Negative (5)	Negative (5)
Aikawa T et al., 2022 [89]	NR	NR	24.00	NA	NR	NR	1	Negative (1)	NR
Alania-Torres E et al., 2021 [90]	NR	NR	NR	NR	NR	NR	NR	Negative (1)	Negative (1)
Albert E et al., 2021 [88]	NR	NR	26.40	NA	18.94	NA	1	Negative (1)	Negative (1)
Ammirati E et al., 2021 [87]	NR	NA	29.00	NA	NR	NR	1	Negative (1)	Negative (1)
Aviram G et al., 2022 [86]	NR	NR	301.25	121.30	NR	NR	NR	Negative (4)	Negative (4)
Bautista Garcia J et al., 2021 [85]	NR	NR	NR	NR	NR	NR	1	Negative (1)	Negative (1)
Bengel C et al., 2022 [84]	NR	NR	32.00	17.25	NR	NR	2	Negative (2)	NR
Bews H et al., 2022 [83]	NR	NR	43.00	38.01	NR	NR	9	NR	NR
Cereda A et al., 2021 [79]	NR	NR	24.00	NA	6.53	NA	1	Negative (1)	Negative (1)
Chachar T et al., 2021 [80]	NR	NR	70.00	NA	2.10	NA	1	Negative (1)	Negative (1)
Chamling B et al., 2021 [81]	NR	NR	13.20	NA	NR	NR	NR	Negative (2)	NR
Chellapandian SB et al., 2022 [82]	NR	NR	6.10	NA	NR	NR	NR	Negative (1)	Negative (1)
Chow BT and Lai CK, 2022 [77]	NR	NR	NR	NR	NR	NR	1	NR	NR
Cimaglia P et al., 2022 [78]	NR	NR	19.00	NA	NR	NR	1	Negative (1)	Negative (1)
D’Angelo T et al., 2021 [76]	NR	NR	39.60	NA	12.56	NA	1	Negative (1)	Negative (1)
Deb A et al., 2021 [75]	41	NA	155.00	NA	NR	NR	1	Negative (1)	Negative (1)
Diaz GA et al., 2021 [74]	NR	NR	NR	NR	NR	NR	NR	Negative (20)	Negative
Dickey JB et al., 2021 [73]	NR	NR	NR	NR	12.60	6.80	3	Negative (3)	NR
Dlewati M et al., 2022 [72]	21	NA	51.00	NA	4.40	NA	1	Negative (1)	Negative (1)
Gautam N et al., 2021 [71]	40	NA	NR	NR	4.96	NR	NR	Negative (1)	Negative (1)
Habedank D et al., 2022 [70]	NR	NR	NR	NR	NR	NR	1	NR	NR
Habib MB et al., 2021 [69]	NR	NR	NR	NR	NR	NR	1	Negative (1)	Negative (1)
Jahnke C et al., 2022 [68]	NR	NR	NR	NR	NR	NR	2	NR	NR
Kaneta K et al., 2022 [67]	NR	NR	NR	NR	NR	NR	1	NR	NR
Kaul R et al., 2021 [66]	17.5	3.53	49.00	15.56	12.38	6.54	2	Negative (2)	Negative (2)
Kawakami T et al., 2022 [65]	NR	NR	17.80	NA	NR	NR	1	Negative (1)	Negative (1)
Kim HW et al., 2021 [64]	16	12.49	48.67	23.13	2.34	NA	4	Negative (3)	Negative (2)
Kim IC et al., 2021 [61]	27	NA	77.00	NA	2.28	NA	1	NR	Negative (1)
King WW et al., 2021 [63]	NR	NR	68.00	50.88	10.73	9.48	4	NR	Negative (3)
Koizumi T et al., 2021 [60]	NR	NR	NR	NR	NR	NR	2	Negative (2)	NR
Korosoglou G et al., 2022 [62]	NR	NR	71.00	NA	NR	NR	0	Negative (1)	NR
Larson KF et al., 2021 [59]	NR	NR	6.59	4.05	NR	NR	8	Negative (8)	NR
Lee CH and Kong EJ, 2022 [58]	NR	NR	NR	NR	5.99	NA	1	Negative (1)	NR
Levin D et al., 2021 [56]	NR	NR	300.64	41.58	NR	NR	7	NR	NR
Maeda M et al., 2022 [19]	NR	NR	NR	NR	18.40	NA	1	Negative (1)	Negative (1)
Maki H et al., 2022 [54]	NR	NR	10.70	NA	8.80	NA	1	NR	NR
Manfredi R et al., 2022 [57]	NR	NR	58.00	21.20	NR	NR	2	NR	NR
Mansour J et al., 2021 [55]	16	12.73	16.50	12.02	8.15	8.27	2	Negative (3)	Negative (2)
Marhshall M et al., 2021 [16]	40	NA	127.00	NA	NR	NR	1	Negative (1)	Negative (1)
Matta A et al., 2021 [53]	7	NA	44.20	NA	0.25	NA	1	NR	NR
Mengesha B et al., 2022 [52]	NR	NR	68.00	NA	NR	NR	1	Negative (1)	Negative (1)
Meyer-Szary J et al., 2022 [51]	NR	NR	39.00	NA	NR	NA	1	NR	NR
Miqdad MA et al., 2021 [46]	18	NA	42.00	NA	4.50	NA	1	NR	NR
Montgomery J et al. 2021 [50]	NR	NR	NR	NR	NR	NR	23	Negative (19), Not performed (4)	Negative (13), Not Performed (10)
Mouch S et al., 2021 [49]	NR	NR	65.80	22.70	NR	NR	4	Negative (4)	Negative (4)
Murakami Y et al., 2022 [48]	NR	NR	1.30	0.00	NR	NR	2	Negative (1)	Negative (2)
Muthukumar A et al., 2021 [47]	25	NA	19.10	NR	NR	NR	1	NR	NR
Nagasaka T et al., 2022 [45]	NR	NR	101.60	NA	4.55	NR	NR	Negative (1)	Negative (1)
Nevet A et al., 2021 [38]	NR	NR	NR	NR	NR	NR	NR	NR	NR
Nguyen TD et al., 2021 [37]	NR	NR	19.60	NA	NR	NR	1	Negative (1)	Negative (1)
Nunn S et al., 2022 [44]	NR	NR	12.00	NA	NR	NR	1	Negative (1)	NR
Ohnishi M et al., 2022 [43]	NR	NR	75.70	NA	NR	nR	1	Negative (1)	Negative (1)
Onderko L et al., 2021 [42]	21.5	3.54	59.15	13.36	NR	NR	3	Negative (3)	NR
Parmar K et al., 2022 [41]	17.5	7.77	85.47	51.71	NR	NR	NR	NR	NR
Patel YR et al., 2021 [40]	25.5	5.00	85.00	31.00	37.40	16.60	5	Negative (5)	Negative (5)
Patrignani A et al., 2021 [39]	NR	NR	NR	NR	NR	NR	1	Negative (1)	NR
Sano M et al., 2022 [36]	NR	NR	19.00	NA	NR	NR	2	Negative (1)	Negative (1)
Schmitt P et al., 2021 [29]	NR	NR	59.00	NA	NR	NR	1	Negative (1)	Positive for VCA-IgG (1)
Shaw KE et al., 2021 [35]	NR	NR	NR	NR	6.46	2.12	2	NR	NR
Shiyovich A et al., 2022 [34]	NR	NR	NR	NR	1.34	2.42	4	NR	NR
Shumkova M et al., 2021 [33]	NR	NR	79.00	NA	NR	NR	1	Negative (1)	Negative (1)
Singh B et al., 2021 [32]	21	NA	24.80	NA	NR	NR	1	Negative (1)	Negative (1)
Sokolska J et al., 2021 [31]	NR	NR	82.00	NA	NR	NR	1	Negative (1)	Negative (1)
Starekova J et al., 2021 [30]	NR	NR	NR	NR	5.26	4.82	4	Negative (4)	NR
Tailor P et al., 2021 [22]	12	NA	63.50	NA	NR	NR	1	Negative (1)	NR
Verma A et al., 2021 [23]	22	22.63	26.80	NA	16.57	14.75	2	Negative (2)	Negative (1), NR (1)
Vidula MK et al., 2021 [24]	27.5	2.12	4.61	3.97	NR	NR	2	Negative (2)	Negative (2)
Viskin D et al., 2021 [21]	NR	NR	NR	NR	NR	NR	8	Negative (8)	Negative (7)
Watkins K et al., 2021 [28]	NR	NR	NR	NR	NR	NR	NR	Negative (1)	NR
Williams CB et al., 2021 [27]	NR	NR	111.00	NA	NR	NR	1	Negative (1)	NR
Wong J et al., 2022 [26]	45	NA	50.90	NA	NR	NR	1	Negative (1)	Negative (1)
Wu B et al., 2022 [25]	19	NA	76.00	NA	NR	NR	1	Negative (1)	Negative (1)
Yen KC et al., 2022 [20]	NR	NR	44.47	NA	2.21	NA	1	Negative (1)	Negative (1)

CRP: C-reactive protein, cTnI: Cardiac troponin I, ESR: Erythrocyte sedimentation rate, NA: Not applicable, NR: Not reported, SD: Standard deviation.

**Table 5:** Clinical Course and Common Treatment Details of Patients [16,19,20,21,22,23,24,25,26,27,28,29,30,31,32,33,34,35,36,37,38,39,40,41,42,43,44,45,46,47,48,49,50,51,52,53,54,55,56,57,58,59,60,61,62,63,64,65,66,67,68,69,70,71,72,73,74,75,76,77,78,79,80,81,82,83,84,85,86,87,88,89,90,91,92].

Author, Year	Length of Hospital Stay (Days)		ED Admission (n)	ICU Admission (n)	Treatment								
	Mean	SD			Steroids	Colchicine	NSAIDs	Paracetamol	Antibiotics	Beta-Blockers	ACE Inhibitors	Anti Platelets	Anti Coagulants
Abellan C et al., 2021 [92]	4	1.00	3	NR	0	0	1	0	0	0	3	0	1
Ahmed SK, 2022 [91]	2.6	1.14	NR	0	0	3	5	0	0	0	0	0	0
Aikawa T et al., 2022 [89]	3	NA	NR	NR	0	0	0	0	0	0	0	0	0
Alania-Torres E et al., 2021 [90]	9	NA	0	0	0	1	2	1	0	0	0	0	0
Albert E et al., 2021 [88]	NR	NR	1	NR	0	0	0	0	0	1	0	0	0
Ammirati E et al., 2021 [87]	7	NA	1	NR	0	NR	0	NR	NR	NR	NR	NR	NR
Aviram G et al., 2022 [86]	NR	NR	NR	NR	NR	NR	NR	NR	NR	NR	NR	NR	NR
Bautista Garcia J et al., 2021 [85]	6	NA	1	NR	NR	NR	1	NR	NR	NR	NR	NR	NR
Bengel C et al., 2022 [84]	6	0.00	2	NR	0	1	0	0	0	1	1	0	0
Bews H et al., 2022 [83]	4.22	1.30	NR	NR	0	6	7	0	0	0	0	0	0
Cereda A et al., 2021 [79]	7	NA	1	0	0	0	0	0	1	1	1	0	0
Chachar T et al., 2021 [80]	2	NA	NR	NR	0	0	0	0	0	1	1	0	0
Chamling B et al., 2021 [81]	NR	NR	2	NR	0	0	1	0	0	0	0	0	1
Chellapandian SB et al., 2022 [82]	NR	NR	1	NR	0	1	1	0	0	0	0	0	0
Chow BT and Lai CK, 2022 [77]	NR	NR	2	NR	1	0	0	0	0	1	1	1	1
Cimaglia P et al., 2022 [78]	7	NA	1	NR	0	0	1	0	0	0	0	0	0
D’Angelo T et al., 2021 [76]	NR	NR	1	NR	1	0	1	0	0	1	0	0	0
Deb A et al., 2021 [75]	NR	NR	NR	NR	0	0	0	0	2	0	0	0	0
Diaz GA et al., 2021 [74]	2	0.71	NR	2	0	9	15	0	NR	NR	NR	0	0
Dickey JB et al., 2021 [73]	NR	NR	NR	NR	NR	NR	NR	NR	NR	NR	NR	NR	NR
Dlewati M et al., 2022 [72]	2	NA	1	NR	0	0	0	0	0	1	1	0	0
Gautam N et al., 2021 [71]	NR	NR	NR	NR	NR	NR	NR	NR	NR	NR	NR	NR	NR
Habedank D et al., 2022 [70]	5	NA	1	NR	NR	NR	NR	NR	NR	NR	NR	NR	NR
Habib MB et al., 2021 [69]	6	NA	1	1	0	0	0	1	0	1	0	1	1
Jahnke C et al., 2022 [68]	6	0.00	NR	0	0	0	2	0	0	0	1	0	1
Kaneta K et al., 2022 [67]	9	NA	NR	NR	NR	NR	NR	NR	NR	NR	NR	NR	NR
Kaul R et al., 2021 [66]	3	0.00	2	NR	1	1	0	0	0	1	0	0	0
Kawakami T et al., 2022 [65]	7	NA	NR	NR	NR	NR	NR	NR	NR	NR	NR	NR	NR
Kim HW et al., 2021 [64]	NR	NR	NR	NR	1	3	2	0	0	0	0	0	0
Kim IC et al., 2021 [61]	5	NA	1	NR	NR	NR	NR	NR	NR	NR	NR	NR	NR
King WW et al., 2021 [63]	2.5	1.00	NR	NR	NR	NR	NR	NR	NR	NR	NR	NR	NR
Koizumi T et al., 2021 [60]	4	NA	1	NR	0	0	2	0	0	0	0	0	0
Korosoglou G et al., 2022 [62]	NR	NR	1	NR	0	0	0	0	0	1	0	0	0
Larson KF et al., 2021 [59]	NR	NR	NR	3	2	2	3	1	NR	NR	NR	NR	NR
Lee CH and Kong EJ, 2022 [58]	5	NR	1	0	0	0	1	0	0	0	0	0	0
Levin D et al., 2021 [56]	3.43	1.40	NR	NR	0	4	2	0	0	4	3	0	0
Maeda M et al., 2022 [19]	NR	NR	1	NR	0	0	0	0	0	0	0	0	0
Maki H et al., 2022 [54]	NR	NR	NR	NR	0	0	0	0	0	1	1	0	0
Manfredi R et al., 2022 [57]	NR	NR	NR	NR	0	2	2	0	0	0	0	0	0
Mansour J et al., 2021 [55]	2	1.41	NR	NR	NR	NR	0	NR	NR	1	NR	NR	NR
Marhshall M et al., 2021 [16]	4	NA	1	0	2	0	2	0	0	0	0	0	0
Matta A et al., 2021 [53]	1	NA	1	0	0	0	1	0	0	0	0	0	0
Mengesha B et al., 2022 [52]	NR	NR	NR	NR	1	0	0	0	1	1	1	0	0
Meyer-Szary J et al., 2022 [51]	9	NA	NR	NR	0	0	0	1	0	0	0	0	0
Miqdad MA et al., 2021 [46]	7	NA	1	0	0	1	1	0	0	0	1	0	0
Montgomery J et al. 2021 [50]	NR	NR	NR	NR	NR	NR	NR	NR	NR	NR	NR	NR	NR
Mouch S et al., 2021 [49]	6	1.83	NR	NR	0	4	4	0	0	0	0	0	0
Murakami Y et al., 2022 [48]	9	0.00	1	0	0	2	2	0	0	0	0	0	0
Muthukumar A et al., 2021 [47]	4	NA	1	NR	0	0	0	0	0	1	1	0	0
Nagasaka T et al., 2022 [45]	NR	NR	NR	NR	0	0	1	0	0	0	0	0	0
Nevet A et al., 2021 [38]	NR	NR	3	0	0	3	3	0	0	0	0	0	0
Nguyen TD et al., 2021 [37]	NR	NR	1	0	NR	NR	NR	NR	NR	NR	NR	NR	NR
Nunn S et al., 2022 [44]	4	NA	0	0	0	0	0	0	0	1	0	0	0
Ohnishi M et al., 2022 [43]	3	NA	NR	1	0	0	0	1	0	0	0	0	0
Onderko L et al., 2021 [42]	NR	NR	NR	NR	0	1	1	0	0	3	0	0	0
Parmar K et al., 2022 [41]	7.5	2.89	NR	NR	4	4	4	0	0	0	0	0	0
Patel YR et al., 2021 [40]	1.8	0.45	5	0	0	4	0	0	0	1	1	0	0
Patrignani A et al., 2021 [39]	NR	NR	1	NR	0	NR	0	NR	NR	NR	NR	NR	NR
Sano M et al., 2022 [36]	6	NA	0	0	NR	NR	NR	NR	NR	NR	NR	NR	NR
Schmitt P et al., 2021 [29]	NR	NR	0	0	NR	NR	NR	NR	NR	NR	NR	NR	NR
Shaw KE et al., 2021 [35]	NR	NR	NR	NR	NR	NR	NR	NR	NR	NR	NR	NR	NR
Shiyovich A et al., 2022 [34]	NR	NR	NR	NR	NR	NR	NR	NR	NR	NR	NR	NR	NR
Shumkova M et al., 2021 [33]	6	NA	1	NR	1	0	1	0	1	0	0	0	0
Singh B et al., 2021 [32]	4	NA	1	NR	NR	NR	NR	NR	NR	NR	NR	NR	NR
Sokolska J et al., 2021 [31]	NR	NR	NR	NR	NR	NR	NR	NR	NR	NR	NR	NR	NR
Starekova J et al., 2021 [30]	NR	NR	4	NR	NR	NR	NR	NR	NR	NR	NR	NR	NR
Tailor P et al., 2021 [22]	5	NA	NR	NR	0	1	0	0	0	1	1	0	0
Verma A et al., 2021 [23]	5	2.83	NR	NR	1	1	1	0	2	0	0	1	2
Vidula MK et al., 2021 [24]	NR	NR	2	0	NR	NR	NR	NR	NR	NR	NR	NR	NR
Viskin D et al., 2021 [21]	NR	NR	8	NR	NR	NR	NR	NR	NR	NR	NR	NR	NR
Watkins K et al., 2021 [28]	NR	NR	1	NR	0	1	0	0	0	0	0	0	0
Williams CB et al., 2021 [27]	5	NA	NR	NR	0	1	1	0	0	1	1	0	0
Wong J et al., 2022 [26]	2	NA	1	NR	0	0	1	0	0	1	0	0	0
Wu B et al., 2022 [25]	4	NA	1	NR	1	0	0	0	0	0	0	0	0
Yen KC et al., 2022 [20]	5	NA	1	NR	0	0	0	0	0	0	0	0	0

ACE: Angiotensin-converting enzyme, ED: Emergency department, ICU: Intensive care unit, NA: Not applicable, NR: Not reported, NSAID: Non-steroidal anti-inflammatory drug, SD: Standard deviation.

## Data Availability

With publication, the data set used for this meta-analysis will be shared upon request from the study authors.

## Supplementary Materials

The following supporting information can be downloaded at: https://www.mdpi.com/article/10.3390/jcm11154521/s1, Table S1: Methodological Quality Assessment [16,19,20,21,22,23,24,25,26,27,28,29,30,31,32,33,34,35,36,37,38,39,40,41,42,43,44,45,46,47,48,49,50,51,52,53,54,55,56,57,58,59,60,61,62,63,64,65,66,67,68,69,70,71,72,73,74,75,76,77,78,79,80,81,82,83,84,85,86,87,88,89,90,91,92]; Supplementary Item S1: Search strategy; Supplementary Item S2: Flow diagram for study selection [130].
