# Supplementary material for: Clinical Characteristics of Patients with Myocarditis following COVID-19 mRNA Vaccination: A Systematic Review and Meta-Analysis
Source: J Clin Med. 2022 Aug 3;11(15):4521. doi: 10.3390/jcm11154521 (PMC9369856; doi:10.3390/jcm11154521)
Supplement: Supplementary file 1 [file jcm-11-04521-s001.zip › Supplementary Item S1 Search-Strategy.pdf]

Supplementary Item S1. Search Strategy

OVID

Database(s): **Embase** 1988 to 2021 Week 27**, Ovid MEDLINE(R) 1946 to Present and Epub Ahead of Print, In-Process & Other Non-Indexed Citations and Ovid MEDLINE(R) Daily, EBM Reviews - Cochrane Central Register of Controlled Trials** June 2021**, EBM Reviews - Cochrane Database of Systematic Reviews** 2005 to July 8, 2021

Search Strategy:

#	Searches
1	((("corona-vir*" or coronavi* or "corona vir*" or covid* or hcov*) adj5 ("19" or "2019" or nouveau or nuevo or wuhan or hubei or china or chinese or pandemic* or outbreak or shutdown or "shut down*" or "shut-down*" or quarantin* or (lock* adj down) or "lock-down*" or lockdown* or "stay-at-home" or "shelter-in-place")) or ((pneumon* or "severe-acute-respiratory-syndrome" or sars*) adj3 (hubei or wuhan)) or (2019novelcov or "2019-novel cov" or 19ncov* or "19-ncov*" or 2019ncov* or "2019-ncov" or covid19* or covid2019* or "covid-19*" or "covid-2019*" or "covid 2019*" or "covid 19*" or cov19* or cov2019* or "cov-19*" or "cov-2019*" or "corona virinae19" or "corona virinae2019" or "corona-virinae19" or "corona-virinae2019" or coronavirinae19 or coronavirinae2019 or "coronavirinae-19" or "coronavirinae-2019" or "corona virus19" or " corona-virus19" or "corona virus2019" or "corona-virus2019" or coronavirus19 or coronavirus2019 or "coronavirus-19" or "coronavirus-2019" or ("corona virus" adj1 disease adj2 ("19" or "2019")) or (("Corona virinae" or "corona virus" or Coronavirinae or coronavirus or COVID* or nCoV*) and (wuhan or hubei)) or hcov19 or hcov2019 or "hcov-19" or hcov2019 or "hcov-2019" or ncoron* or ncov* or "new coronavirus*" or "novel coronavirus*" or "SARS corona virus 2" or "SARS corona-virus 2" or "SARS-coronavirus2" or "SARS-coronavirus-2" or "SARS-COV-2" or "SARS-COV2" or "SARSCOV-2" or "SARSCOV 2" or "Severe Acute Respiratory Syndrome Corona virus 2" or "Severe Acute Respiratory Syndrome Coronavirus 2" or "Severe Acute Respiratory Syndrome cov 2" or "Severe Acute Respiratory Syndrome cov2" or betacoronavirus or wn-cov* or wncov*)).ti,ab,hw,kw. or (((exp Coronavirus/ or exp Coronavirus Infections/ or exp coronavirinae/) and ("19" or "2019" or nouveau or nuevo or wuhan or hubei or china or chinese or pandemic* or outbreak or shutdown or "shut down*" or "shut-

	down*" or quarantin* or (lock* adj down) or "lock-down*" or lockdown* or "stay-at-home" or "shelter-in-place").ti.) or *pandemics/) or covid*.ti.
2	(Severe Acute Respiratory Syndrome Coronavirus 2 or COVID-19 or COVID-19 drug treatment or COVID-19 serotherapy or COVID-19 diagnostic testing or COVID-19 vaccine or spike glycoprotein, COVID-19 virus).os,ps,rs,ox,px,rx,nm.
3	COVID-19/ or SARS-CoV-2/
4	((mRNA* adj "1273") or "mRNA-1273" or (Ad26* adj CoV2*) or (NVX* adj CoV2373) or "BNT162b2" or RBD or BBV152 or tozinameran or (Pfizer* adj1 BioNTech) or moderna or astrazeneca or "oxford-astrazeneca" or (johnson adj johnson)).ti,ab,hw,kw.
5	or/1-4
6	(vaccine* or vaccinat*).ti,ab,hw,kw.
7	exp Vaccines/
8	exp *Vaccination/
9	or/6-8
10	Myocarditis/ or (myocarditis or pericarditis).ti,ab,kw,kf. or (cardiac* or heart or "chest pain" or angina or coronary or troponin*).ti.
11	5 and 9 and 10
12	(conference abstract or conference review or editorial or erratum or note or addresses or autobiography or bibliography or biography or blogs or comment or dictionary or directory or interactive tutorial or interview or lectures or legal cases or legislation or news or newspaper article or patient education handout or periodical index or portraits or proceedings or published erratum or video-audio media or webcasts).mp. or conference abstract.st.
13	11 not 12
14	(exp animals/ or exp nonhuman/) not exp humans/
15	((alpaca or alpacas or amphibian or amphibians or animal or animals or antelope or armadillo or armadillos or avian or baboon or baboons or beagle or beagles or bee or bees or bird or birds or bison or bovine or buffalo or buffaloes or buffalos or "c elegans" or "Caenorhabditis elegans" or camel or camels or canine or canines or carp or cats or cattle or chick or chicken or chickens or chicks or chimp or chimpanze or chimpanzees

	or chimps or cow or cows or "D melanogaster" or "dairy calf" or "dairy calves" or deer or dog or dogs or donkey or donkeys or drosophila or "Drosophila melanogaster" or duck or duckling or ducklings or ducks or equid or equids or equine or equines or feline or felines or ferret or ferrets or finch or finches or fish or flatworm or flatworms or fox or foxes or frog or frogs or "fruit flies" or "fruit fly" or "G mellonella" or "Galleria mellonella" or geese or gerbil or gerbils or goat or goats or goose or gorilla or gorillas or hamster or hamsters or hare or hares or heifer or heifers or horse or horses or insect or insects or jellyfish or kangaroo or kangaroos or kitten or kittens or lagomorph or lagomorphs or lamb or lambs or llama or llamas or macaque or macaques or macaw or macaws or marmoset or marmosets or mice or minipig or minipigs or mink or minks or monkey or monkeys or mouse or mule or mules or nematode or nematodes or octopus or octopuses or orangutan or "orang-utan" or orangutans or "orang-utans" or oxen or parrot or parrots or pig or pigeon or pigeons or piglet or piglets or pigs or porcine or primate or primates or quail or rabbit or rabbits or rat or rats or reptile or reptiles or rodent or rodents or ruminant or ruminants or salmon or sheep or shrimp or slug or slugs or swine or tamarin or tamarins or toad or toads or trout or urchin or urchins or vole or voles or waxworm or waxworms or worm or worms or xenopus or "zebra fish" or zebrafish) not (human or humans or patient or patients)).ti,ab,hw,kw.
16	(rat or rats or mice or mouse or murine or pig or pigs or porcine or swine or dog or dogs).ti.
17	or/14-16
18	13 not 17
19	limit 18 to yr="2019 -Current"
20	limit 19 to english language [Limit not valid in CDSR; records were retained]
21	limit 19 to no language specified [Limit not valid in CDSR; records were retained]
22	20 or 21
23	remove duplicates from 22

SCOPUS

1	(TITLE-ABS-KEY ((corona-vir* OR "corona vir*" OR coronavir* OR covid* OR hcov*) W/5 ("19" OR "2019" OR novel OR new OR nuevo OR nouveau OR wuhan OR hubei)) OR TITLE-ABS-KEY (covid19 OR covid2019 OR ncov* OR "sars corona virus 2" OR "sars coronavirus 2" OR "sars-cov-2*" OR "sarscov-2*" OR sars-cov2* OR "Severe Acute Respiratory Syndrome Corona virus 2" OR "Severe Acute Respiratory Syndrome Coronavirus 2") OR TITLE-ABS-KEY (corona-virinae19 OR corona-virinae2019 OR corona-virus19 OR corona-virus2019 OR "corona virus19" OR "corona virus2019" OR coronavirinae19 OR coronavirinae2019 OR "corona virinae19" OR "corona virinae2019" OR coronavirus19 OR coronavirus2019 OR covid19 OR covid2019 OR ncov* OR hcov* OR betacoronavirus OR "beta-coronavirus" OR wn-cov* OR wncov*) OR TITLE-ABS-KEY (pneumonia W/2 wuhan) OR TITLE (((coronavr* OR coronovir* OR corona-vir* OR corono-vir* OR "corona vir*" OR "corono vir*") W/3 (pandemic OR shutdown OR "shut down*" OR "shut-down*" OR quarantin* OR disease OR (lock* W/ down) OR "lock-down*" OR lockdown*)) OR covid*))
2	TITLE-ABS-KEY (myocarditis or pericarditis) OR TITLE (cardiac* or heart or "chest pain" or angina or coronary or troponin*)
3	TITLE-ABS-KEY ((vaccine* or vaccinat*))
4	1 and 2
5	INDEX(embase) OR INDEX(medline) OR PMID(0* OR 1* OR 2* OR 3* OR 4* OR 5* OR 6* OR 7* OR 8* OR 9*)
6	4 not 5
7	(TITLE-ABS-KEY ((alpaca OR alpacas OR amphibian OR amphibians OR animal OR animals OR antelope OR armadillo OR armadillos OR avian OR baboon OR baboons OR beagle OR beagles OR bee OR bees OR bird OR birds OR bison OR bovine OR buffalo OR buffaloes OR buffalos OR "c elegans" OR "Caenorhabditis elegans" OR camel OR camels OR canine OR canines OR carp OR cats OR cattle OR chick OR chicken OR chickens OR chicks OR chimp OR chimpanze OR chimpanzees OR chimps OR cow OR cows OR "D melanogaster" OR "dairy calf" OR "dairy calves" OR deer OR dog

	OR dogs OR donkey OR donkeys OR drosophila OR "Drosophila melanogaster" OR duck OR duckling OR ducklings OR ducks OR equid OR equids OR equine OR equines OR feline OR felines OR ferret OR ferrets OR finch OR finches OR fish OR flatworm OR flatworms OR fox OR foxes OR frog OR frogs OR "fruit flies" OR "fruit fly" OR "G mellonella" OR "Galleria mellonella" OR geese OR gerbil OR gerbils OR goat OR goats OR goose OR gorilla OR gorillas OR hamster OR hamsters OR hare OR hares OR heifer OR heifers OR horse OR horses OR insect OR insects OR jellyfish OR kangaroo OR kangaroos OR kitten OR kittens OR lagomorph OR lagomorphs OR lamb OR lambs OR llama OR llamas OR macaque OR macaques OR macaw OR macaws OR marmoset OR marmosets OR mice OR minipig OR minipigs OR mink OR minks OR monkey OR monkeys OR mouse OR mule OR mules OR nematode OR nematodes OR octopus OR octopuses OR orangutan OR "orang-utan" OR orangutans OR "orang-utans" OR oxen OR parrot OR parrots OR pig OR pigeon OR pigeons OR piglet OR piglets OR pigs OR porcine OR primate OR primates OR quail OR rabbit OR rabbits OR rat OR rats OR reptile OR reptiles OR rodent OR rodents OR ruminant OR ruminants OR salmon OR sheep OR shrimp OR slug OR slugs OR swine OR tamarin OR tamarins OR toad OR toads OR trout OR urchin OR urchins OR vole OR voles OR waxworm OR waxworms OR worm OR worms OR xenopus OR "zebra fish" OR zebrafish) AND NOT (human OR humans OR patient OR patients)))
8	6 not 7
9	PUBYEAR AFT 2018 AND LIMIT-TO (LANGUAGE , "English")
10	8 and 9
