# Supplementary material for: Clinical Characteristics of Patients with Myocarditis following COVID-19 mRNA Vaccination: A Systematic Review and Meta-Analysis
Source: J Clin Med. 2022 Aug 3;11(15):4521. doi: 10.3390/jcm11154521 (PMC9369856; doi:10.3390/jcm11154521)
Supplement: Supplementary file 1 [file jcm-11-04521-s001.zip › Supplementary Item S2 Flow diagram for study selection.pdf]

PRISMA 2009 Flow Diagram

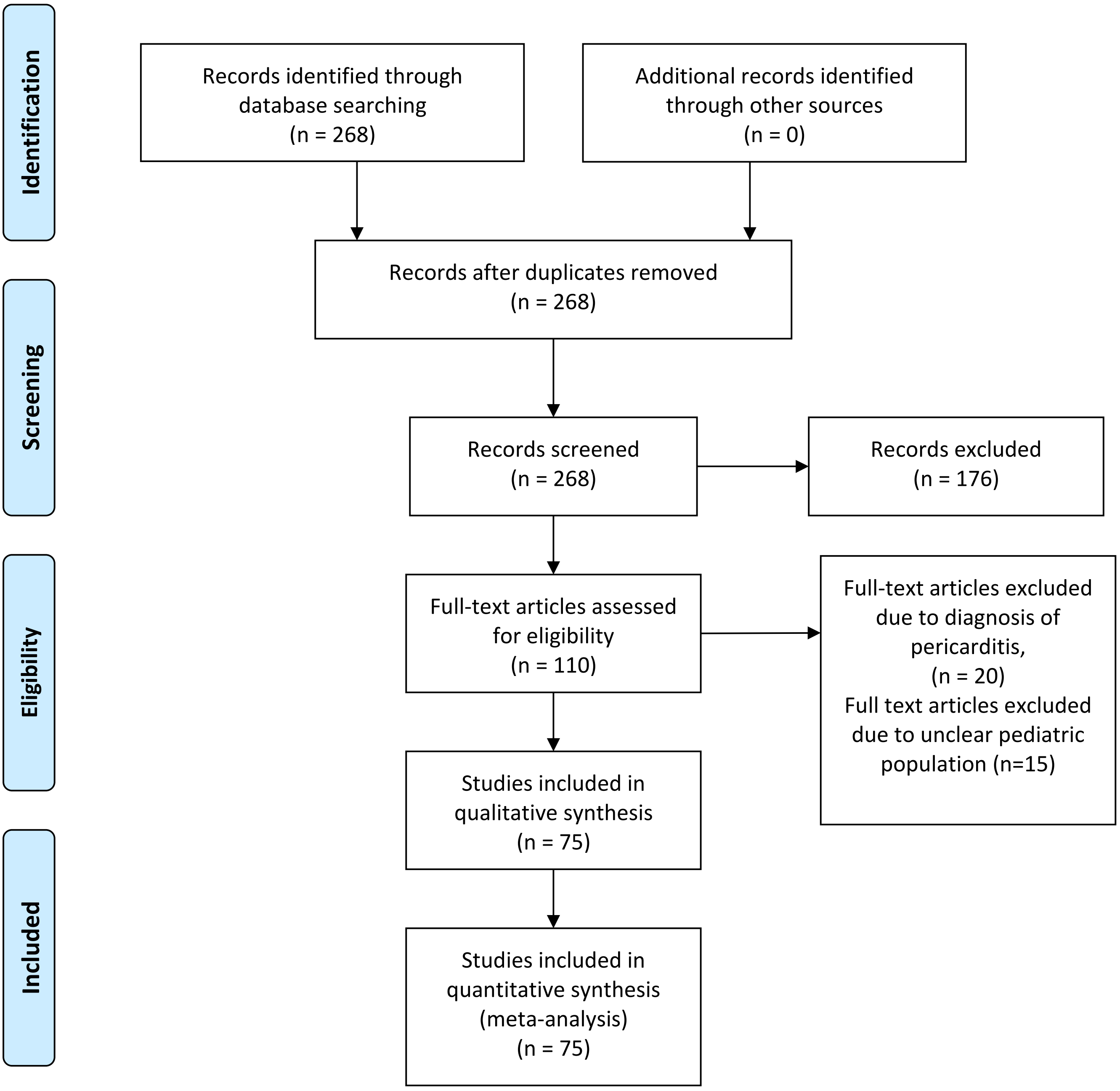

From: Moher D, Liberati A, Tetzlaff J, Altman DG, The PRISMA Group (2009). *P*referred *R*eporting *I*tems for *S*ystematic Reviews and *M*eta-*A*nalyses: The PRISMA Statement. PLoS Med 6(7): e1000097. doi:10.1371/journal.pmed1000097

For more information, visit www.prisma-statement.org.
