# Supplementary material for: Clinical Characteristics of Patients with Myocarditis following COVID-19 mRNA Vaccination: A Systematic Review and Meta-Analysis
Source: J Clin Med. 2022 Aug 3;11(15):4521. doi: 10.3390/jcm11154521 (PMC9369856; doi:10.3390/jcm11154521)
Supplement: Supplementary file 1 [file jcm-11-04521-s001.zip › Table S1.pdf]

Supplementary Table S1 Methodological Quality Assessment

Author, Year	Question 1	Question 2	Question 3	Question 4	Question 5
Abellan C et al., 2021 [92]	Yes	Yes	Yes	Yes	Yes
Ahmed SK, 2022 [91]	Yes	Yes	Yes	No	Yes
Aikawa T et al., 2022 [89]	Yes	Yes	Yes	Yes	Yes
Alania-Torres E et al., 2021 [90]	Yes	Yes	Yes	Yes	Yes
Albert E et al., 2021 [88]	Yes	Yes	Yes	Yes	Yes
Ammirati E et al., 2021 [87]	Yes	Yes	Yes	No	Yes
Aviram G et al., 2022 [86]	Yes	Yes	Yes	Yes	Yes
Bautista Garcia J et al., 2021 [85]	Yes	Yes	Yes	Yes	Yes
Bengel C et al., 2022 [84]	Yes	Yes	Yes	No	Yes
Bews H et al., 2022 [83]	Yes	No	Yes	Yes	Yes
Cereda A et al., 2021 [79]	Yes	Yes	Yes	No	Yes
Chachar T et al., 2021 [80]	Yes	Yes	Yes	Yes	Yes
Chamling B et al., 2021 [81]	Yes	No	Yes	No	Yes

Chellapandian SB et al., 2022 [82]	Yes	Yes	Yes	Yes	Yes
Chow BT and Lai CK, 2022 [77]	Yes	Yes	Yes	No	Yes
Cimaglia P et al., 2022 [78]	Yes	Yes	Yes	Yes	Yes
D'Angelo T et al., 2021 [76]	Yes	Yes	Yes	Yes	Yes
Deb A et al., 2021 [75]	Yes	Yes	Yes	No	Yes
Diaz GA et al., 2021 [74]	Yes	Yes	Yes	No	Yes
Dickey JB et al., 2021 [73]	Yes	Yes	No	Yes	Yes
Dlewati M et al., 2022 [72]	Yes	Yes	Yes	No	Yes
Gautam N et al., 2021 [71]	Yes	Yes	Yes	Yes	Yes
Habedank D et al., 2022 [70]	Yes	Yes	Yes	Yes	Yes
Habib MB et al., 2021 [69]	Yes	Yes	Yes	Yes	Yes
Jahnke C et al., 2022 [68]	Yes	Yes	Yes	No	Yes
Kaneta K et al., 2022 [67]	Yes	Yes	Yes	No	Yes
Kaul R et al., 2021 [66]	Yes	Yes	Yes	Yes	Yes
Kawakami T et al., 2022 [65]	Yes	Yes	Yes	Yes	Yes
Kim HW et al., 2021 [64]	Yes	Yes	Yes	Yes	Yes

Kim IC et al., 2021 [61]	Yes	Yes	Yes	Yes	Yes
King WW et al., 2021 [63]	Yes	Yes	Yes	Yes	Yes
Koizumi T et al., 2021 [60]	Yes	Yes	Yes	Yes	Yes
Korosoglou G et al., 2022 [62]	Yes	Yes	Yes	Yes	Yes
Larson KF et al., 2021 [59]	Yes	Yes	Yes	No	Yes
Lee CH and Kong EJ, 2022 [58]	Yes	Yes	Yes	No	Yes
Levin D et al., 2021 [56]	Yes	Yes	Yes	Yes	Yes
Maeda M et al., 2022 [19]	Yes	Yes	Yes	Yes	Yes
Maki H et al., 2022 [54]	Yes	Yes	Yes	Yes	Yes
Manfredi R et al., 2022 [57]	Yes	Yes	Yes	Yes	Yes
Mansour J et al., 2021 [55]	Yes	Yes	Yes	Yes	Yes
Marhshall M et al., 2021 [16]	Yes	Yes	Yes	No	Yes
Matta A et al., 2021 [53]	Yes	Yes	Yes	Yes	Yes
Mengesha B et al., 2022 [52]	Yes	Yes	Yes	No	Yes
Meyer-Szary J et al., 2022 [51]	Yes	Yes	Yes	Yes	Yes
Miqdad MA et al., 2021 [46]	Yes	Yes	Yes	No	Yes

Montgomery J et al. 2021 [50]	Yes	Yes	Yes	Yes	Yes
Mouch S et al., 2021 [49]	Yes	Yes	Yes	Yes	Yes
Murakami Y et al., 2022 [48]	Yes	Yes	Yes	Yes	Yes
Muthukumar A et al., 2021 [47]	Yes	Yes	Yes	Yes	Yes
Nagasaka T et al., 2022 [45]	Yes	Yes	No	No	Yes
Nevet A et al., 2021 [38]	Yes	Yes	Yes	Yes	Yes
Nguyen TD et al., 2021 [37]	Yes	Yes	Yes	Yes	Yes
Nunn S et al., 2022 [44]	Yes	Yes	Yes	Yes	Yes
Ohnishi M et al., 2022 [43]	Yes	Yes	Yes	No	Yes
Onderko L et al., 2021 [42]	Yes	Yes	Yes	Yes	Yes
Parmar K et al., 2022 [41]	Yes	Yes	Yes	Yes	Yes
Patel YR et al., 2021 [40]	Yes	Yes	Yes	Yes	Yes
Patrignani A et al., 2021 [39]	Yes	Yes	Yes	Yes	Yes
Sano M et al., 2022 [36]	Yes	Yes	Yes	Yes	Yes
Schmitt P et al., 2021 [29]	Yes	Yes	Yes	Yes	Yes
Shaw KE et al., 2021 [35]	Yes	Yes	Yes	No	Yes

Shiyovich A et al., 2022 [34]	Yes	Yes	Yes	Yes	Yes
Shumkova M et al., 2021 [33]	Yes	Yes	Yes	Yes	Yes
Singh B et al., 2021 [32]	Yes	Yes	Yes	No	Yes
Sokolska J et al., 2021 [31]	Yes	Yes	Yes	No	Yes
Starekova J et al., 2021 [30]	Yes	Yes	Yes	Yes	Yes
Tailor P et al., 2021 [22]	Yes	Yes	Yes	Yes	Yes
Verma A et al., 2021 [23]	Yes	Yes	Yes	Yes	Yes
Vidula MK et al., 2021 [24]	Yes	Yes	Yes	Yes	Yes
Viskin D et al., 2021 [21]	Yes	Yes	Yes	Yes	Yes
Watkins K et al., 2021 [28]	Yes	Yes	Yes	Yes	Yes
Williams CB et al., 2021 [27]	Yes	Yes	Yes	Yes	Yes
Wong J et al., 2022 [26]	Yes	Yes	Yes	Yes	Yes
Wu B et al., 2022 [25]	Yes	Yes	Yes	Yes	Yes
Yen KC et al. 2022 [20]	Yes	Yes	Yes	Yes	Yes

Question 1: Does the patient(s) represent(s) the whole experience of the investigator (centre)

Question 2: Was the exposure adequately ascertained?

Question 3: Was the outcome adequately ascertained?

Question 4: Was follow-up long enough for outcomes to occur?

Question 5: Is the case(s) described with sufficient details to allow other investigators to replicate the research or to allow practitioners make inferences related to their own practice?
